# Effect of Short- to Long-Term Exposure to Ambient Particulate Matter on Cognitive Function in a Cohort of Middle-Aged and Older Adults: KoGES

**DOI:** 10.3390/ijerph19169913

**Published:** 2022-08-11

**Authors:** Jane J. Lee, Ji Hyun Kim, Dae Sub Song, Kyoungho Lee

**Affiliations:** Division of Population Health Research, Department of Precision Medicine, Korea National Institute of Health, Korea Disease Control and Prevention Agency, 200 Osongsaengmyeong 2-ro, Osong-eup, Heungdeok-gu, Cheongju-si 28160, Chungcheongbuk-do, Korea

**Keywords:** air pollution, particulate matter, cognitive function, cohort study, epidemiology, PM_2.5_, PM_10_, MMSE

## Abstract

Exposure to ambient air pollution and its threat to human health is a global concern, especially in the elderly population. Therefore, more in-depth studies are required to understand the extent of the harmful effects of particulate matter (PM) based on duration and levels of exposure. An investigation was conducted to determine the association between short- (1–14 days), medium- (1, 3, and 6 months), and long-term (1, 2, and 3 years) exposure to air pollutants (PM_2.5_ and PM_10_) and cognitive function among Koreans (4175 participants, mean age 67.8 years, 55.2% women) aged over 50 years. Higher levels of PM_2.5_ exposure for short to long term and PM_10_ exposure for medium to long term were found to be associated with decreased cognitive function, as indicated by lower scores of the Mini-Mental State Examination adopted in Korean (K-MMSE). There were significant effect modifications by sex, age group, alcohol consumption, physical activity, and smoking status in the association between long-term PM_2.5_ and PM_10_ exposure and cognitive function. These findings, which underscore the importance of the efforts to reduce the exposure levels and durations of air pollutants, especially in the vulnerable elderly population, provide evidence for establishing more stringent policies for air pollution regulations.

## 1. Introduction

Air pollution, which is a critical global concern due to its threat to public health and welfare [1], is regarded as one of the leading causes of disease based on an estimated 79 risk factors in 195 countries from 1990 to 2015 [2]. Chronic exposure to outdoor air pollution contributes to the pathogenesis of major cardiovascular and respiratory diseases [3] via the direct and indirect (i.e., triggering oxidative stress and activating inflammatory pathways) actions of particles on the cardiovascular system [4]. Among the various types of air pollutants, airborne particulate matter (PM), a heterogeneous combination of particles of different sizes and types, including chemical compounds, has been extensively studied for its adverse health-related effects [5]. The PM types are further classified based on the diameter, of which PM_10_ includes particles with a diameter of 10 µm or less, while PM_2.5_ includes particles with diameters of 2.5 µm or less [6]. Although particles ≤10 µm are inhaled into the lungs, PM_2.5_ has been postulated to pose more adverse health effects due to its ability to further penetrate the lower parts of the respiratory system, such as small bronchi and pulmonary alveoli, and also due to presence of heavy metals and carcinogens [7,8]. Despite rapid industrialization and urbanization in South Korea, the government has made tremendous efforts to lower PM levels over the decades [9]. However, evidence has demonstrated that even lower levels of PM exposure are associated with decreased cognitive function, suggesting the need to further lower the concentration of ambient PM [10].

In 2020, the elderly population aged 60 years and older was reported to comprise 15.7% of the total population (51,829,136) in Korea [11]. The proportion of the elderly population is expected to increase to 46.4% by 2070 [11]. With a rapidly increasing elderly population, maintaining cognitive function until later age has become the aim for healthy aging. To achieve this, identifying the risk factors for cognitive impairment is crucial for delaying the onset and speed of cognitive decline. As a modifiable risk factor, there has been an increased interest in investigating the harmful effects of air pollution on cognitive function in the elderly population.

Understanding the degree of adverse health effects attributed to air pollution across various regions and demographics is crucial because the extent of these effects is highly dependent upon the sources and compositions of PM and the duration of exposure, as well as the susceptibility, vulnerability, and characteristics of the exposed population [12]. Therefore, more in-depth studies are required to understand the extent of the harmful effects of PM based on the duration and levels, and the types of exposure that can be translated in the Korean population, particularly in the vulnerable middle-aged-to-elderly population. Importantly, there is a need for a better proactive response to air pollution, especially among vulnerable aging populations. 

We hypothesized that higher exposure to PM is associated with decreased cognitive function. With respect to the duration of exposure, we hypothesized that long-term exposure to PM is more closely related to cognitive performance than short- or medium-term PM exposures. Among PM_2.5_ and PM_10_ exposures, we further hypothesized that the association would be more prominent with PM_2.5_ compared to PM_10_ exposure. The present study investigated the association between short-, medium-, and long-term exposure to ambient PM, including PM_2.5_ and PM_10_, on cognitive function among Korean Genome and Epidemiology Study (KoGES) Ansan and Ansung participants over 50 years of age.

## 2. Materials and Methods

### 2.1. Study Population

The KoGES is a large prospective population-based study initiated in the early 2000s by Korean government entities to investigate public health issues [13]. The sample in this study comprised subjects from the Ansan and Ansung study who attended the seventh follow-up (eighth examination) in 2015–2016 and participated in an aging sub-study. The Ansan and Ansung study recruited participants from urban (Ansan) and rural (Ansung) areas to collect data from two distinctive community-dwelling populations. Detailed information on the KoGES Ansan and Ansung study, including design and methodology, has been previously described [13]. 

**Figure 1:**
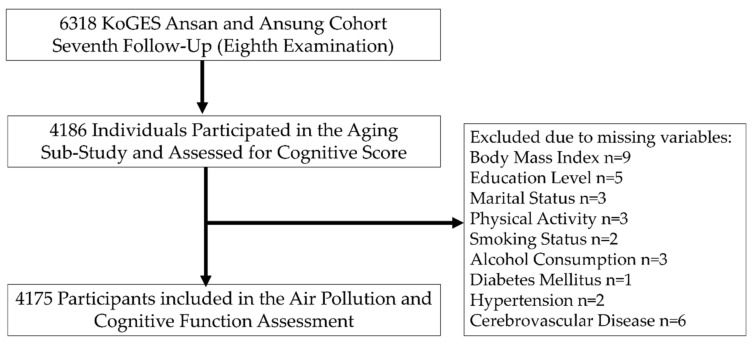
Flowchart of study method.

In the present investigation, we used the most recent and largest follow-up data available for air pollution (PM_2.5_ and PM_10_) and cognitive assessment scores. A flowchart of the study participants is shown in Figure 1. Among 6318 participants from the seventh follow-up, 2132 individuals had not participated in the aging sub-study and thus were excluded from the cognitive test. An additional 11 participants were excluded because of missing covariates, such as body mass index (BMI) (n = 9), education level (n = 5), marital status (n = 3), physical activity (n = 3), smoking (n = 2), alcohol consumption (n = 3), diabetes mellitus (n = 1), hypertension (n = 2), and cerebrovascular disease (n = 6), resulting in a total of 4175 participants available for investigation. The study protocol was approved by the Institutional Review Board of the Korea National Institute of Health and the Korea Centers for Disease Control and Prevention. All participants provided their written consent.

### 2.2. Cognitive Function Assessment

The Mini-Mental State Examination (MMSE) is one of the most widely used tools to assess cognitive impairment by evaluating mental status. The MMSE is also commonly used to screen individuals for possible dementia in primary care settings [14]. The MMSE adopted in the study was in the Korean language (K-MMSE), and it has been previously validated and administered by trained interviewers [15]. The K-MMSE comprises seven items that assess cognitive function in the following dimensions: orientation, memory and registration, attention and calculation, recall, and language. The K-MMSE scores range from 0 to 30, with higher scores indicating better cognitive performance. The continuous MMSE score was dichotomized using a conventional cutoff of 24 points [16], i.e., participants with a K-MMSE score of 24 points or higher were classified as having a normal cognitive function, while those with a score ≤23 were categorized as having decreased cognitive function. 

### 2.3. Ambient Particulate Matters

The ambient PM_2.5_ and PM_10_ levels before the study visit of individual levels were collected. Depending on the duration of PM exposure, we categorized short-, medium-, and long-term exposures as follows:

1. Short-term air pollution exposure data included PM_2.5_ and PM_10_ exposures on the date of the visit (day 1), and the average of 2-, 3-, 4-, 5-, 6-, 7-, 8-, 9-, 10-, 11-, 12-, 13-, and 14-day counting from the date of each participant’s study visit;
2. Medium-term air pollution exposure data included average PM_2.5_ and PM_10_ exposure levels over 1, 3, and 6 months;
3. Long-term air pollution exposure data included average PM_2.5_ and PM_10_ exposures over 1, 2, and 3 years.

Air quality parameters (e.g., PM_2.5_ and PM_10_) and meteorological databases (e.g., humidity and temperature) were established using the Community Multiscale Air Quality (CMAQ) model, which is based on a chemical transport model. Of note, no PM measurement methods were used in this study to assess air quality data. After quantifying the PM_2.5_ and PM_10_ concentrations every 1 km^2^ (1 km × 1 km) and the meteorological data every 81 km^2^ (9 km × 9 km), air quality data were generated based on a geocoding method, where the local exposure data were tabulated by regressing the gridded data as a weighted sum based on the border of the city, county, and district. Air quality and meteorological data were linked to the study participants’ data according to the date of the study visit and participants’ residential addresses. A more detailed methodology for the generation and linkage of air quality and meteorological data has been described previously [17,18]. 

### 2.4. Potential Covariates

Potential covariates were selected from the review of the literature associated with exposure to air pollution and cognitive function. In the KoGES Ansan and Ansung eighth examination (seventh follow-up), trained interviewers asked a series of questions to ascertain information regarding demographic characteristics, socioeconomic status, medical history, health-related lifestyle behaviors (e.g., smoking status, alcohol consumption), and use of medications. 

BMI was calculated using participants’ weight in kg and height in m^2^ (kg/m^2^). Seated systolic and diastolic blood pressures at rest were measured three times and the average of three measurements were used. Diabetes mellitus was defined as fasting plasma glucose ≥ 126 mg/dL, hemoglobin A1c ≥ 6.5%, diagnosis of diabetes mellitus, and/or use of insulin or oral hypoglycemic medications. Hypertension was defined as systolic blood pressure ≥ 140 mmHg, diastolic blood pressure ≥ 90 mmHg, diagnosis of hypertension, and/or use of anti-hypertensive medication. Hyperlipidemia was defined as having at least one of the following conditions: high cholesterol, high triglycerides, low high-density lipoprotein (HDL), diagnosed with hyperlipidemia, or taking lipid-lowering medication. Cerebrovascular disease was defined as at least one of the following conditions: myocardial infarction, congestive heart failure, coronary artery disease, and/or cerebrovascular disease. 

### 2.5. Statistical Analysis

Descriptive statistics, including means and standard deviations (SDs) for continuous variables and frequencies and percentages for categorical variables, were used to summarize participant characteristics of cognitive function on exposure to ambient air pollutants. For ambient PM levels and K-MMSE scores, Pearson correlation coefficients adjusted with and without age were computed to explore the relationships between PM and cognitive function. 

For the primary analysis, the associations between short-, medium-, and long-term exposures to PM and cognitive function were assessed using a (1) multivariable-adjusted linear regression model for continuous K-MMSE scores and (2) multivariable-adjusted logistic regression model for dichotomous K-MMSE outcomes (normal cognitive function vs. decreased cognitive function), while each PM level was included as a dependent variable in a separate statistical model.

The β-coefficients from the linear regression models were based on an interquartile range increase at each PM level, while the odds ratios from the logistic regression models were based on a one-unit increase in each PM level. 

The list of potential covariates included age in years, sex (women or men), BMI in kg/m^2^, educational level (below elementary school, elementary school, middle and high school, college and above), marital status (married, separated or divorced, widowed, others (e.g., single, living together)), geographical region (Ansan or Ansung), physical activity (yes or no as to whether the study participants regularly exercise to make their bodies sweat at least once a week), smoking (never, former, current), alcohol consumption (never, former, moderate, heavy), diabetes mellitus (yes or no), hypertension (yes or no), hyperlipidemia (yes or no), cerebrovascular disease (yes or no), prior diagnosis of cancer (yes or no), relative temperature in °C, relative humidity in %, and season of participants’ study visit. We included relative temperature and humidity, as well as the season of the study visit, based on their relationship with PM concentrations documented in the literature [19,20].

To avoid multicollinearity, the variation inflation factor (VIF) was examined before including potential covariates in the statistical model. Variables with a VIF greater than 10 were removed from the list of covariates and were not adjusted in the statistical models. The humidity, temperature, and season of the study visit were incorporated into the statistical models because of their direct relationships with ambient air pollutants. 

Linearity tests for the association between PM and cognitive function were performed based on fitting semiparametric generalized additive models while adjusting the identical list of covariates described above [21]. Each semiparametric generalized additive model included the PM variable as a spline smoothing spline term and other variates as strict linear terms. To assess the potential non-linear association, models using the tertiles of each PM exposure level were constructed to examine their association with cognitive function, while comparing the lowest PM exposure group (tertile 1 as a reference) with intermediate (tertile 2) and highest (tertile 3) PM exposure groups.

As a secondary analysis, multivariable-adjusted linear analysis with a forward selection procedure was performed to identify the sets of variables that predicted cognitive function the most. Each PM level was entered into a separate model, with variables added to the model based on their significance level (*p*-value cutoff of 0.5). The list of variables included in the model was the same as the potential covariates included in the primary analysis, except for relative temperature, humidity, and season, which were included in the primary analysis because of their relationship with air pollutants.

We assessed the statistical significance of the potential effect modifiers by including a term for the interaction between PM exposures and each of the following variable in the multivariable-adjusted linear model: sex, age group (50–65 years, 65–75 years, >75 years), alcohol consumption, physical activity, and smoking status. Further, we performed stratified subgroup analyses according to the potential effect modifiers. 

A two-tailed *p*-value of less than 0.05 was considered statistically significant. Statistical analyses were conducted using SAS software (version 9.4; SAS Institute, Cary, NC, USA).

## 3. Results

### Study Sample Characteristics

Descriptive statistics provided in Table 1 reveal the demographic, physical, clinical, and lifestyle characteristics of the 4175 study participants. The distribution of all ambient PM levels and K-MMSE scores was approximately normal, with skewness of ±2 and kurtosis of ±7. Among the 4175 participants, the mean age (SD) was 67.8 (7.9) years, with a range of 52–82 years. Of the group, 55.2% were women, while the mean BMI (SD) was 24.5 (3.3) kg/m^2^, with 40.9% of participants classified as obese (i.e., BMI of 25 kg/m^2^ or higher). More than three-quarters of the participants were married (n = 3293), while 36.5% had completed middle and high school. Hypertension showed the highest prevalence rate (52.7%), followed by hyperlipidemia (43.9%) and diabetes mellitus (22.0%). Cerebrovascular disease was least prevalent (7.2%), followed by cancer (11.7%). A total of 926 of 4175 (22.2%) participants were classified as having decreased cognitive function (K-MMSE score of 23 or less).

The distributions of PM_2.5_ and PM_10_ levels according to the duration of exposure (short, medium, and long term) are shown in Table 2. The average exposure concentrations of both PM_2.5_ and PM_10_ gradually increased from short to long term, reaching a peak at the 3-year average. As the PM levels generally decreased over the years of this study population, the 3-year average of PM_2.5_ and PM_10_ levels exhibited the highest value. The average (SD) 3-year exposures to PM_2.5_ and PM_10_ were 28.7 (4.4) µg/m^3^ and 52.4 (3.5) µg/m^3^, respectively.

The results of the unadjusted and age-adjusted partial Pearson correlations between PM exposure and cognitive scores are shown in Table 3. Simple Pearson correlations indicated that short-, medium-, and long-term exposures to PM_2.5_ were negatively associated with cognitive scores, ranging from −0.35 to −0.06, whereas only medium- to long-term exposures (average of 10 to 14 days, average of 1 and 6 months, and average of 1, 2, and 3 years) to PM_10_ were negatively associated with cognitive scores, ranging from −0.34 to −0.03. This trend of association generally persisted when these correlations were adjusted for age. Several exceptions include the following: 1-day PM_2.5_ value and short-term PM_10_ exposures became non-significant. 

For continuous cognitive scores as an outcome, the associations between various PM exposures and cognitive scores are presented in Table 4. All potential covariates listed in Section 2 were included in the multivariable-adjusted model, except for geographical location, because of a high VIF of greater than 10. Most of the short-, medium-, and long-term exposures to PM_2.5_ were negatively associated with cognitive scores, confirming that higher exposure to PM_2.5_, regardless of the exposure duration, was associated with decreased cognitive function. For instance, an IQR increase (9.4 µg/m^3^) in 3-year average PM_2.5_ was associated with a 1.16-point decrease in the K-MMSE score. In addition, the higher concentrations of short-term exposure to PM_2.5_ were significantly associated with cognitive scores, except for the day of the study visit (i.e., 1-day) and the 2-day average. For PM_10_ exposure, the 1–12-day average concentrations were not associated with cognitive function. With no exceptions, associations with cognitive score were seen for 13-day average exposure and after for an IQR increase in PM_10_, and this included all of the medium- to long-term exposures to PM_10_.

Significant nonlinear associations were detected by fitting semiparametric models for 8-day (*p* = 0.02), 10-day (*p* = 0.02), and 3-year (*p* = 0.04) average exposure levels of PM_2.5_ on cognitive score (P for linearity, Table 4). Multivariable-adjusted regression and logistic analyses were repeated using tertiles of PM exposure levels (Supplementary Tables S1 and S2). As listed in Supplementary Table S2, the analysis by tertiles of PM levels further showed that intermediate and high levels of exposure to short-, medium-, and long-term PM_2.5_ (tertile 2 and 3) were closely associated with decreased cognitive function, as compared to the lowest (tertile 1) exposure to PM_2.5_, suggesting the importance of the level of exposure. For 2-year average PM_2.5_, a high exposure level of PM_2.5_ (tertile 3) was associated with a 0.76-point decrease in K-MMSE score compared with those who had the lowest level of PM_2.5_ exposure (tertile 1). For medium- to long-term exposure to PM_10_, intermediate (tertile 2) to high (tertile 3) levels of PM_10_ exposure were more closely associated with lower K-MMSE scores compared to the lowest PM_10_ exposure (tertile 1).

For cognitive function as a dichotomous variable (normal vs. decreased cognitive function), the relationships between the various durations of PM exposure and cognitive function are shown in Supplementary Table S1. Consistent with the results derived from the multivariable-adjusted linear regression models, short-, medium-, and long-term exposure to higher levels of PM_2.5_ and medium- to long-term exposure to PM_10_ were associated with decreased cognitive function. For PM_2.5_, except for the 1-day PM exposure level, higher short-term exposure levels were associated with decreased cognitive function, whereas most of the short-term exposure to PM_10_ was not associated with decreased cognitive function. Similar trends were noted in the analysis according to the tertiles of PM_2.5_ and PM_10_. 

Forward selection regression analyses identified a subset of variables predictive of cognitive function (Table 5). Variables that reflect short-, medium-, and long-term exposure to PM_2.5_ were selected by this procedure, except for the 1-day PM_2.5_ exposure level. For PM_10_, only medium- to long-term exposure to ambient air pollution was included in the selection model.

The model consistently selected educational level, age, physical activity, diabetes mellitus, marital status, and smoking status. Based on the results of the multivariable-adjusted regression model, older age and diabetes were associated with decreased cognitive function. Being married, exercising regularly, and having a higher educational level were generally associated with higher cognitive function (data not shown). Depending upon the type of air pollution data in different periods, smoking status was entered in the model in the 6th or 7th order, and the current-smoker group was associated with decreased cognitive function. BMI was also selected when long-term exposure to PM was considered in the pool of model selection. The R^2^ value ranged from 0.37 to 0.39, confirming that this list of variables explains 37–39% of the variance in cognitive function.

Results from the multivariable-adjusted linear regression analyses with the interaction term between PM and selected potential effect modifiers on cognitive score, and the subgroup analysis stratified by each group, are shown in Supplementary Tables S3–S7. The interaction terms were significant for sex, age group, alcohol consumption, physical activity, and smoking status with long-term exposure to ambient PM_2.5_ and PM_10_ on cognitive score. Based on the stratified analysis, we found that the adverse effect of long-term PM exposure on cognitive function was higher in (1) women compared to men, (2) >75 years group compared to younger age groups, (3) non-drinkers compared to former or current drinkers, (4) non-exercise group compared to exercise group, and (5) never smokers compared to current or former smokers. 

## 4. Discussion

### 4.1. Principal Findings

The current study explored the associations between ambient PM exposure levels under various durations, including short-, medium-, and long-term exposures to PM_2.5_ and PM_10_, and cognitive function in community-dwelling, middle-aged-to-elderly adults in South Korea. Our principal findings are 4-fold. First, higher exposure to ambient PM_2.5_ and PM_10_ concentrations during the medium- to long-term period was associated with decreased cognitive function, even after adjusting for multiple covariates that are known to be associated with air pollution exposure and cognitive function. Second, between PM_2.5_ and PM_10_, only higher exposure to PM_2.5_ concentrations during the short term was associated with decreased cognitive performance after accounting for various covariates, confirming the differences in the association of PM_2.5_ and PM_10_ on cognitive function during the short-term exposure period. Third, this study found several factors that were significantly associated with cognitive function, including PM exposure, education level, age, physical activity, diabetes mellitus, marital status, and smoking status. BMI was also selected when long-term air pollution data were considered in the model. Fourth, significant effect modifications of sex, age group, alcohol consumption, physical activity, and smoking status were observed in the association between long-term PM_2.5_ and PM_10_ exposure and cognitive function. Overall, these findings add to the growing body of literature showing that medium- and long-term exposure to ambient PM is associated with cognitive decline in middle-aged and older adults in Korea. To the best of our knowledge, this is the first study to investigate the association of ambient PM_2.5_ and PM_10_ exposure under various durations of short, medium, and long term on cognitive function in middle-aged-to-elderly populations in Korea for a relatively large sample size of 4175 participants. 

### 4.2. In the Context of the Current Literature

The current literature lacks consistency in reporting the association between air pollution exposure and cognitive performance, presumably due to the differences in participants’ characteristics, exposure levels of ambient PM, the methodology and techniques in assessing PM, duration of PM exposure, study designs, outcomes, and covariates [22,23,24]. Therefore, a direct comparison between our results and those of prior studies requires caution. However, when compared with the studies that assessed cognitive function using the MMSE, the trend of the association is favorable in supporting the evidence of the harmful effect of PM on cognitive function. A study of 1054 Taiwanese participants over 60 years of age reported a significant association between yearly estimated exposure to PM_2.5_ and decreased MMSE score (i.e., decreased cognitive function; *p* = 0.039), and a relationship between yearly exposure to PM_10_ and decreased MMSE score assessing the subdomain of the cognitive score, including language, construction, and obey (*p* = 0.013) [25]. The mean level of PM_2.5_ and PM_10_ exposures in this Taiwanese population was higher than the exposure levels in the current study (35.2 vs. 27.7 for PM_2.5_ and 62.9 vs. 50.6 for PM_10_ for the Taiwanese study vs. the current study, respectively). Another prospective study of 13,324 Chinese participants with a mean age of 82.4 years assessed the relationship between average annual PM_2.5_ exposure between 2002 and 2014 and the incidence of poor cognitive function determined using the Chinese version of the MMSE. This study suggested that long-term exposure to PM_2.5_, as well as 3-year average exposure to PM_2.5,_ were associated with an increased risk of poor cognitive function [26]. Among the studies in South Korea, a study of 1484 elderly participants detected gender differences, with women showing a higher risk of decreased cognitive function assessed by the K-MMSE when exposed to PM_10_; however, no main effect was observed between PM_10_ exposure and cognitive function [13]. Another Korean study of 2896 participants older than 70 years of age reported a significant association between the yearly assessed levels of PM_2.5_, PM_10_, and the Korean version of the MMSE, which was consistent with our findings. Our study results add to the growing body of evidence that higher exposure to ambient PM_2.5_ during the short, medium, and long term, as well as PM_10_ exposure during the medium to long term, are associated with an adverse effect on cognitive function in the middle-aged and older Korean population.

The exposure levels of ambient PM_2.5_ and PM_10_ observed in our study population were lower than those reported in China [20], India [27], and Pakistan [28], higher than the levels reported in the United States (US) and Canada [29,30], and within similar ranges as reported in France [31]. Zhou et al. reported the ranges of ambient PM in China using 589 pairs of data encompassing 57 urban and rural cities and regions [20]. The ranges of ambient PM were ~60–100 µg/m^3^ for PM_2.5_ and 100–170 µg/m^3^ for PM_10_ across urban and rural areas in northern and southern China [20]. These ranges are at least twice as high as those observed in the current dataset assessed in South Korea. Owing to the different levels of exposure to ambient PM, the adverse effects of air pollution may differ depending on the geographical location of the individuals.

Based on the National Ambient Air Quality Standards (NAAQS) established by the US Environmental Protection Agency (EPA), the primary standard for PM_2.5_ should not exceed 12.0 µg/m^3^ annually and 35 µg/m^3^ based on a 24 h average, and the 24 h primary standard for PM_10_ does not exceed 150 µg/m^3^ more than once per year on average over a 3-year period. The Korean air quality standard is slightly different, with a 24 h standard of 35 µg/m^3^ for PM_2.5_ and 100 µg/m^3^ for PM_10_. The annual standard is 15 µg/m^3^ for PM_2.5_ and 50 µg/m^3^ for PM_10_ [32]. The World Health Organization (WHO) 2021 global air quality guidelines provide the most stringent levels, with a 24 h standard of 15 µg/m^3^ for PM_2.5_ and 45 µg/m^3^ for PM_10_, and an annual standard of 5 µg/m^3^ for PM_2.5_ and 15 µg/m^3^ for PM_10_. The annual levels of PM_2.5_ and PM_10_ reported in the current study exceeded the Korean air quality standards, whereas the 24 h levels of PM_2.5_ and PM_10_ levels were lower than the Korean standards [33]. The lack of association between 1-day PM_2.5_ level and short-term exposure to PM_10_ on cognitive function may be due to the lower levels of ambient PM concentrations that the study participants were exposed to. Notably, the overall PM exposure levels of the current study participants exceeded the WHO air quality guidelines, which provide the most conservative levels. Therefore, the study sample included in the current analysis may be at high risk of the harmful effects of air pollution due to the high levels of long-term PM exposure. 

This study included a list of covariates that are associated with cognitive function. It is well established by the current literature that the male sex [34], education [35], marriage [36,37], and regular exercise [38] are associated with better cognitive function. In contrast, higher BMI [39], smoking [40], excessive alcohol consumption [41], diabetes mellitus [42], elevated blood pressure [43], cerebrovascular disease [44], and cancer treatment or conditions [45] are related to decreased cognitive function. The results of our forward selection regression (Table 5) model identified several factors from this pool of well-established variables and mirrored the findings from previous studies. Of note, prior studies have explored the underlying mechanisms of these factors. For instance, differences in the type and level of sexual hormone secretions, as well as in the brain structure and functions, may explain the influence of sex on cognitive function [46]. In addition, educational attainment may be causally related to cognitive development, thus affecting cognitive function. 

Smoking is a modifiable behavioral factor that can significantly affect health. The association between smoking and cognitive health has been documented extensively, with the evidence suggesting that smoking cessation reduces the associated adverse risks [47,48]. In the context of ambient PM exposure and cognitive function, smoking status as an effect modifier showed different aspects of the association, e.g., never smokers, rather than smokers or quitters, were more prone to the adverse effects of ambient PM exposure [49]. The stratified analysis based on smoking status in this study revealed that PM exposure was related to a decrease in cognitive function among the never-smoker group, which is consistent with the prior finding by Yao et al. [49]. Our study found that most of the associations in former- and current-smoker groups were insignificant. Never smokers may be more vulnerable to the adverse effect of ambient PM exposure as they have not been directly exposed to substances, viz. tobacco, that harm the human body; the bodily reactions and cognitive abilities of never-smokers may be less active than those of smokers or quitters.

Participants from the current study were recruited from two distinctive regions, with one characterized by an urban area (Ansan) and the other by a rural area (Ansung). The two regions had different PM_2.5_ and PM_10_ levels, with the rural area having higher levels of both PM_2.5_ and PM_10_ during short- to long-term exposure compared to the urban area, which we suspect was influenced by other factors such as the topography of the area and meteorological conditions [50]. The overall regional altitude is higher in Ansung than in Ansan; elevation is closely related to meteorological conditions and, thus, to ambient PM levels. In addition, prior studies have reported that higher PM levels are associated with lower temperature, wind speed, and precipitation due to diffusion and wet deposition [50,51]. Moreover, higher PM is related to lower average relative humidity [51]. Differences in the meteorological data between the two regions were observed, with Ansan having statistically higher levels of average temperature, wind speed, insolation, and surface pressure, and lower relative humidity, which may explain the differences in the PM levels. When the geographical region was entered into the regression model, we found a high VIF between the air pollution data and other variables; thus, the region was removed from the model.

The current study observed that short-, medium-, and long-term exposure to PM_2.5_ was associated with a decreased cognitive score, but only medium- to long-term exposure to PM_10_ was associated with cognitive function. The current study hypothesized that PM_2.5_ would be more closely associated with cognitive function than PM_10_ due to the following reasons: both PM_2.5_ and PM_10_ are inhalable in the lungs; however, due to the smaller size of the particles (2.5 µm or less in diameter), PM_2.5_ is suspected of causing more adverse health issues than PM_10_. Ambient PM carries various contaminants (e.g., dioxins, iron, lead) on their surfaces, which act as vehicles for delivering toxins to the brain, causing direct and indirect damage [12]. Considering its smaller particle size, larger surface area, and slower sedimentation speed, PM_2.5_ is a more suitable carrier for chemical substances than PM_10_ [52]. Importantly, PM_2.5_ is more likely to travel and accumulate on the surface of the deeper parts of the lung, triggering tissue damage and inflammation, whereas PM_10_ is more likely to stay and deposit on the surfaces of the airways, causing less damage [53]. Accordingly, short-term exposure to PM_10_ may not have a significant influence on cognitive function compared with short-term exposure to PM_2.5_. As noted above, in conjunction with the Korean air quality standards, the non-significant association between short-term PM_10_ exposure and cognitive function may be attributed to the lower levels of PM_10_ exposure. 

Emission sources can provide valuable information on ambient PM. Emissions from non-combustion sources are prone to produce disproportionately large and coarse particles that are less likely to enter the deeper side of the lungs or bloodstream than smaller particles [54,55]. In contrast, combustion sources tend to generate large numbers of smaller particles that are biologically active. In addition, emission sources from mobile sources tend to emit particles into the breathing zone instead of aloft through a stack [56]. The smaller particles can enter the bloodstream and brain straight via the olfactory bulbs [57], and they (e.g., ultrafine particles) also tend to coagulate and condense onto larger particles, resulting in the rapid change of the particle size profile. Subsequently, it is essential to know the types of sources and their locations within the study areas. Using particular PM assessment approaches (i.e., the land-use regression model for assessing exposure to vehicle exhaust [58] or the satellite imagery and the Hybrid Single Particle Lagrangian Integrated Trajectory model to classify natural and anthropogenic PM sources [59]) could facilitate the elucidation of the health effects of PM that originate from specific sources [60,61].

### 4.3. Potential Physiological Mechanisms

The physiological mechanism underlying the health effects of ambient PM exposure, particularly on cognitive function, remains unclear. Discussions of the potential mechanisms that may explain our findings are warranted. One explanation is that metals and various compounds present in ambient PMs enter the circulatory system and trigger systemic inflammation and oxidative stress, which further exhibit harmful effects on cognitive function [62,63]. Ambient PM consists of various types of metals, such as aluminum, lead, arsenic, mercury, iron, copper, nickel, chromium, cadmium, manganese, and zinc, and some of these levels exceed the limits of the WHO or the respective country’s air quality standards [64,65]. These heavy metals, which are present at high levels in polluted ambient air, enter the human body via direct inhalation through the mouth and nose/olfactory epithelium, absorption via the exposed skin, and ingestion into the gastrointestinal tract [66]. As confirmed by a rat model exposed to PM_2.5_, heavy metals bound to PM_2.5_ were accumulated in the blood and various organs [67]. For example, aluminum accumulation was significantly increased in the cerebral cortex of PM_2.5_-treated rats [67]. Aluminum is one of the factors suspected to contribute to the pathogenesis of neurodegenerative diseases [68].

Adverse health effects of other components of ambient PM produced by incomplete combustion, such as polycyclic aromatic hydrocarbons (PAHs), dioxins, furans, and dioxin-like polychlorinated biphenyls, have also been extensively documented in the literature [52,69,70]. Rats exposed to PM_2.5_ containing metallic elements and PAHs (e.g., lead, manganese, aluminum, arsenic, acenaphthene, pyrene, naphthalene, acenaphthylene, benzo[b]fluoranthene, and chrysene; all PAHs > 0.1 mg/mL) had impaired spatial learning, memory, inquiring ability, and sensory function, explained by changes in the ultrastructure of mitochondria and myelin sheaths, as well as abnormal expression in the apoptosis-related proteins [71]. Consequently, the mixture of these PM components may have been linked to pathogenesis. In another recent study, PM_2.5_ exposure induced pathological injury (i.e., changes in the values of inflammatory cytokines and oxidative stress factors) and deoxyribonucleic acid (DNA) damage related to micro-ribonucleic acids (microRNAs) and DNA methylation in rats, suggesting the epigenetic modification capacity of PM exposure [66]. The specific mechanisms require further elucidation; however, the key underlying path of PM-induced effects may include inflammation, oxidative stress, mitochondrial dysfunction, microglial activation, disturbed protein homeostasis, epigenetic modification, genotoxicity, and neuronal death, which leads to neurodegeneration [72]. Future studies are warranted to determine the extent of the association between various types of PMs, including nanoparticles, and to clarify the causal mechanisms between PM exposure and cognitive function. 

### 4.4. Strengths and Limitations

The strengths of this study include: (1) a comprehensive list of PM_2.5_ and PM_10_ exposure variables over short-, medium-, and long-term durations; (2) a well-characterized community-dwelling study design based on a relatively large sample of both women and men with an extensive list of covariates; and (3) the merging of air quality data with each participant’s address provided more accurate individual exposure levels of ambient air pollution.

A limitation is the nature of the observational epidemiological study, which limits the causal inferences from our findings. As the PM_2.5_ exposure duration increased from short to long term, higher levels of PM exposure were associated with more decreased K-MMSE scores, implying the adverse health effect of more prolonged PM exposure on cognitive function. A few exceptions were noted where medium-term exposure had larger effect sizes than some of the long-term exposure levels (i.e., 6-month PM_2.5_ exposure and 3-month PM_10_ exposure levels). Although this study adjusted season, relative temperature, relative humidity, and a comprehensive list of factors as covariates in the statistical model, it is possible that some of the unmeasured factors could have led to such results. This study used outdoor PM exposure levels based on the geographic location of the participants’ residences. Our study population was not assessed for indoor PM levels or sources of indoor PM emissions, such as residential heating and cooking. Accordingly, we were unable to consider the indoor air pollution exposure of the participants, which may have resulted in exposure misclassification. A prior study noted that indoor PM level is related to outdoor PM level due to the exchange of air between the two environments; however, indoor PM levels can potentially exceed outdoor PM levels [73,74]. In addition, individual PM exposure is highly dependent on the indoor air environment as people spend most of their time indoors. Thus, both indoor and outdoor PM levels should be considered in upcoming research. Personal particle exposure monitors could be utilized for the assessment of outdoor and indoor PM levels in future studies [75]. This study used PM_2.5_ and PM_10_ as measures of ambient air pollution exposure because these are routinely measured for monitoring purposes and have international and national guidelines for air quality standards. However, much of the current research focuses on even smaller particles known as ultrafine/nanoparticles/PM_0.1_, whose aerodynamic diameter is 0.1 µm (100 nm) or less [46,55]. These ultrafine particles have larger reactive surface areas, greater capacity to carry harmful surface chemicals, higher deposition in the alveoli of the lungs, a complex composition of combustion-derived ultrafine PM, and greater ability to pass into the circulation and thus undergo rapid accumulation in peripheral organs. Accordingly, ultrafine particles are thought to exhibit even more harmful health effects compared with those from coarse or fine particles [46]. Given that PM_10_ and PM_2.5_ contain PM_0.1_, the associations observed in this current study may be partially attributed to PM_0.1_. Our study population was recruited from two regions in South Korea, which were rural (Ansung) and medium-sized urban areas (Ansan), and were older than 50 years; thus, the generalizability of our findings is not applicable to individuals living in regions with different levels of ambient PM exposure and other types of population. In addition, chemical analysis of PM_2.5_ or PM_10_ on monitoring samples was unavailable in the current study. Further, this study did not measure particle number counts by size category of PM, which can provide valuable information regarding the source of pollutants [76]. Therefore, future investigations that use both the levels of PM and the number of particles during short- to long-term periods to explore their association with cognitive functions are warranted.

## 5. Conclusions

In community-dwelling Korean adults over 50 years old, the current study reported that higher levels of PM_2.5_ during short-, medium-, and long-term durations, as well as PM_10_ during the medium- to long-term duration, were associated with decreased cognitive function, even after accounting for risk factor correlates of ambient air pollution and cognitive function. While variables that are known to be associated with cognitive function are considered, exposure to PM was selected as a predictor of cognitive performance, suggesting that exposure to ambient air pollution should also be considered as a risk factor. 

Overall, our results suggest an adverse effect of both PM_2.5_ and PM_10_ during the medium to long term, as well as short-term exposure to PM_2.5_, on the cognitive ability of middle-aged and elderly adults in South Korea. While exposure to air pollution is considered one of the modifiable risk factors, reducing ambient PM requires tremendous effort by multiple levels and types of national and regional government entities. This study underscored the importance of further efforts to reduce the levels and duration of the air pollutant exposures, especially in the vulnerable elderly population, and provided evidence for strengthening policies for air pollution regulations.

## Figures and Tables

**Table 1:** Characteristics of the study participants (n = 4175).

Characteristic		Mean or N	Standard Deviation or %
Age (years)		67.8	7.9
Sex (%)			
	Women	2305	55.2
	Men	1870	44.8
Age Group (%)			
	50–65 years	1665	39.9
	65–75 years	1483	35.5
	Above 75 years	1027	24.6
BMI (kg/m^2^)		24.5	3.3
BMI Group (%) ^a^			
	Underweight	111	2.7
	Normal Weight	1287	30.8
	Overweight	1069	25.6
	Obese	1708	40.9
Marital Status (%)			
	Married	3293	78.9
	Separated or Divorced	96	2.3
	Widowed	764	18.3
	Others (e.g., Single, Living Together)	22	0.5
Education Level (%)			
	Below Elementary School	879	21.1
	Elementary School	1307	31.3
	Middle and High School	1525	36.5
	College and Above	464	11.1
Season (%)			
	Spring	1118	26.8
	Summer	1561	37.4
	Fall	1236	29.6
	Winter	260	6.2
Geographical Location (%)			
	Ansan	1567	37.5
	Ansung	2608	62.5
Physical Activity (%)			
	No	2982	71.4
	Yes	1193	28.6
Alcohol Consumption (%)			
	Never	2356	56.4
	Former	318	7.6
	Moderate Drinker	1166	27.9
	Heavy Drinker	335	8.0
Smoking Status (%)			
	Never	2750	65.9
	Former	1007	24.1
	Current	418	10.0
Diabetes Mellitus (%)			
	No	3255	78.0
	Yes	920	22.0
Hypertension (%)			
	No	1977	47.4
	Yes	2198	52.7
Cerebrovascular Disease			
	No	3876	92.8
	Yes	299	7.2
Hyperlipidemia (%)			
	No	2341	56.1
	Yes	1834	43.9
Cancer (%)			
	No	3688	88.3
	Yes	487	11.7
K-MMSE Score		25.9	3.6
K-MMSE Category (%) ^b^			
	Normal Cognitive Function	3249	77.8
	Decreased Cognitive Function	926	22.2

BMI, Body Mass Index; K-MMSE, Korean version of Mini-Mental State Examination. ^a^ Underweight was defined as BMI less than 18.5 kg/m^2^; normal weight was defined as BMI 18.5 kg/m^2^ or higher and less than 23 kg/m^2^; overweight was defined as BMI 23 kg/m^2^ or higher and less than 25 kg/m^2^; obese was defined as BMI 25 kg/m^2^ or higher. ^b^ Normal cognitive function was defined as MMSE score of 24 or higher; decreased cognitive function was defined as MMSE score of 23 or lower.

**Table 2:** Distribution of PM_2.5_ and PM_10_ levels according to duration of exposure (n = 4175).

Average Values	PM_2.5_ (µg/m^3^)		PM_10_ (µg/m^3^)	
	Mean	Standard Deviation	Mean	Standard Deviation
1-Day	26.0	13.0	47.0	21.3
2-Day	26.0	11.8	46.6	19.0
3-Day	25.9	10.7	46.5	17.5
4-Day	25.9	9.8	46.7	16.6
5-Day	25.9	9.1	46.9	15.4
6-Day	25.9	8.5	47.1	14.6
1-Week	25.9	8.0	47.2	13.9
8-Day	25.8	7.6	47.2	13.3
9-Day	25.7	7.3	47.1	12.7
10-Day	25.7	7.2	47.2	12.5
11-Day	25.7	7.1	47.2	12.4
12-Day	25.7	7.0	47.2	12.4
13-Day	25.7	7.0	47.2	12.4
2-Week	25.8	7.0	47.3	12.4
1-Month	26.3	6.2	48.1	10.8
3-Month	27.2	5.4	50.2	9.2
6-Month	28.2	4.7	52.0	5.9
1-Year	27.7	4.1	50.6	2.6
2-Year	28.1	4.3	51.5	3.0
3-Year	28.7	4.4	52.4	3.5

**Table 3:** Pearson correlations between PM_2.5_ and PM_10_ with cognitive score, with and without age adjustment (n = 4175).

Average Variables	PM_2.5_ (µg/m^3^)				PM_10_ (µg/m^3^)			
	r	*p*-Value	Age-Adjusted r	*p*-Value	r	*p*-Value	Age-Adjusted r	*p*-Value
1-Day	−0.06	0.0002	−0.02	0.24	0.002	0.89	0.01	0.35
2-Day	−0.08	<0.0001	−0.04	0.02	−0.01	0.37	0.0003	0.99
3-Day	−0.10	<0.0001	−0.05	0.001	−0.02	0.17	−0.01	0.74
4-Day	−0.10	<0.0001	−0.06	0.0003	−0.02	0.20	−0.004	0.81
5-Day	−0.11	<0.0001	−0.06	0.0003	−0.02	0.26	−0.002	0.90
6-Day	−0.11	<0.0001	−0.05	0.001	−0.02	0.21	0.002	0.92
1-Week	−0.11	<.0001	−0.05	0.001	−0.02	0.24	0.01	0.70
8-Day	−0.12	<0.0001	−0.06	0.0003	−0.02	0.14	0.01	0.74
9-Day	−0.13	<0.0001	−0.06	<0.0001	−0.03	0.06	0.002	0.91
10-Day	−0.14	<0.0001	−0.06	<0.0001	−0.03	0.04	0.003	0.83
11-Day	−0.14	<0.0001	−0.07	<0.0001	−0.03	0.03	0.004	0.79
12-Day	−0.14	<0.0001	−0.07	<0.0001	−0.04	0.02	0.005	0.77
13-Day	−0.14	<0.0001	−0.07	<0.0001	−0.03	0.02	0.004	0.78
2-Week	−0.14	<0.0001	−0.06	<0.0001	−0.03	0.03	0.01	0.73
1-Month	−0.16	<0.0001	−0.06	0.0002	−0.04	0.02	0.02	0.22
3-Month	−0.18	<0.0001	−0.05	0.002	−0.02	0.22	0.05	0.001
6-Month	−0.22	<0.0001	−0.07	<0.0001	−0.04	0.01	0.04	0.01
1-Year	−0.32	<0.0001	−0.18	<0.0001	−0.29	<0.0001	−0.16	<0.0001
2-Year	−0.35	<0.0001	−0.19	<0.0001	−0.33	<0.0001	−0.18	<0.0001
3-Year	−0.35	<0.0001	−0.19	<0.0001	−0.34	<0.0001	−0.19	<0.0001

**Table 4:** Relationship between PM_2.5_ and PM_10_ and cognitive score (n = 4175).

Average Value		Interquartile Range	Effect Size ^a^	Standard Error ^a^	*p*-Value	95% Confidence Interval		P for Linearity ^b^
PM_2.5_ (µg/m^3^)								
	1-Day	15.7	−0.05	0.06	0.39	−0.16	0.06	0.70
	2-Day	13.6	−0.10	0.05	0.07	−0.20	0.01	0.30
	3-Day	13.0	−0.16	0.06	0.005	−0.28	−0.05	1.00
	4-Day	12.3	−0.20	0.06	0.001	−0.32	−0.09	0.61
	5-Day	11.7	−0.23	0.06	0.0004	−0.35	−0.10	1.00
	6-Day	11.5	−0.24	0.07	0.0004	−0.38	−0.11	0.71
	1-Week	11.2	−0.27	0.07	0.0002	−0.41	−0.13	0.19
	8-Day	10.4	−0.28	0.07	<0.0001	−0.42	−0.14	0.02
	9-Day	10.1	−0.30	0.07	<0.0001	−0.44	−0.16	0.85
	10-Day	9.6	−0.31	0.07	<0.0001	−0.45	−0.18	0.02
	11-Day	9.3	−0.34	0.07	<0.0001	−0.48	−0.21	0.44
	12-Day	9.1	−0.36	0.07	<0.0001	−0.49	−0.22	0.61
	13-Day	9.3	−0.38	0.07	<0.0001	−0.52	−0.24	0.26
	2-Week	9.3	−0.38	0.07	<0.0001	−0.52	−0.24	0.09
	1-Month	8.4	−0.49	0.08	<0.0001	−0.65	−0.32	0.56
	3-Month	7.6	−0.68	0.11	<0.0001	−0.89	−0.46	0.45
	6-Month	7.9	−0.84	0.12	<0.0001	−1.08	−0.60	0.11
	1-Year	7.3	−0.82	0.15	<0.0001	−1.12	−0.52	0.35
	2-Year	8.9	−1.24	0.21	<0.0001	−1.65	−0.82	0.11
	3-Year	9.4	−1.16	0.23	<0.0001	−1.61	−0.70	0.04
PM_10_ (µg/m^3^)								
	1-Day	25.1	0.05	0.06	0.37	−0.06	0.16	0.58
	2-Day	22.0	0.01	0.06	0.86	−0.10	0.12	0.17
	3-Day	20.7	−0.05	0.06	0.39	−0.17	0.06	0.53
	4-Day	19.6	−0.08	0.06	0.19	−0.20	0.04	0.33
	5-Day	18.6	−0.10	0.07	0.14	−0.23	0.03	0.10
	6-Day	17.8	−0.10	0.07	0.17	−0.24	0.04	0.53
	1-Week	17.9	−0.10	0.08	0.18	−0.25	0.05	0.61
	8-Day	18.3	−0.13	0.08	0.12	−0.29	0.03	0.15
	9-Day	18.4	−0.15	0.09	0.10	−0.32	0.03	0.48
	10-Day	17.6	−0.13	0.09	0.14	−0.30	0.04	0.65
	11-Day	17.5	−0.15	0.09	0.09	−0.32	0.02	0.47
	12-Day	17.9	−0.17	0.09	0.06	−0.35	0.01	0.15
	13-Day	18.0	−0.20	0.09	0.03	−0.38	−0.02	0.09
	2-Week	18.2	−0.21	0.09	0.02	−0.39	−0.03	0.12
	1-Month	18.5	−0.41	0.14	0.004	−0.69	−0.13	0.24
	3-Month	16.0	−0.63	0.18	0.001	−0.99	−0.27	0.20
	6-Month	9.3	−0.43	0.15	0.003	−0.72	−0.14	0.40
	1-Year	4.7	−0.46	0.12	0.0002	−0.70	−0.21	0.75
	2-Year	6.1	−0.76	0.16	<0.0001	−1.07	−0.44	0.37
	3-Year	7.2	−0.85	0.18	<0.0001	−1.20	−0.49	0.64

^a^ Effect sizes and standard errors are shown per interquartile range increase for each PM level. ^b^ Linearity tests for the association between PM and cognitive function were performed based on fitting semiparametric generalized additive models.

**Table 5:** Relationship between exposure to PM_2.5_ and PM_10_ and cognitive score as determined by a forward selection model (n = 4175).

Average Variables		Interquartile Range	Effect Size ^a^	Standard Error ^a^	*p*-Value	Adjusted R^2^	Order Entered for PM	Entered Order
PM_2.5_ (µg/m^3^)								
	1-Day	15.69	-	-	-	0.37	Not Entered	Education, Age, Physical Activity, Diabetes, Marital Status, Smoking
	2-Day	13.63	−0.09	0.05	0.07	0.37	7	Education, Age, Physical Activity, Diabetes, Marital Status, Smoking, PM_2.5_
	3-Day	13.02	−0.14	0.05	0.01	0.38	7	Education, Age, Physical Activity, Diabetes, Marital Status, Smoking, PM_2.5_
	4-Day	12.31	−0.17	0.06	0.002	0.37	6	Education, Age, Physical Activity, Diabetes, Marital Status, PM_2.5_, Smoking
	5-Day	11.70	−0.18	0.06	0.002	0.37	6	Education, Age, Physical Activity, Diabetes, Marital Status, PM_2.5_, Smoking
	6-Day	11.52	−0.18	0.06	0.003	0.37	6	Education, Age, Physical Activity, Diabetes, Marital Status, PM_2.5_, Smoking
	1-Week	11.16	−0.18	0.06	0.003	0.37	6	Education, Age, Physical Activity, Diabetes, Marital Status, PM_2.5_, Smoking
	8-Day	10.36	−0.18	0.06	0.002	0.37	6	Education, Age, Physical Activity, Diabetes, Marital Status, PM_2.5_, Smoking
	9-Day	10.11	−0.20	0.06	0.001	0.37	6	Education, Age, Physical Activity, Diabetes, Marital Status, PM_2.5_, Smoking
	10-Day	9.59	−0.20	0.06	0.001	0.38	6	Education, Age, Physical Activity, Diabetes, Marital Status, PM_2.5_, Smoking
	11-Day	9.33	−0.21	0.06	0.0003	0.37	6	Education, Age, Physical Activity, Diabetes, Marital Status, PM_2.5_, Smoking
	12-Day	9.04	−0.21	0.06	0.0002	0.37	6	Education, Age, Physical Activity, Diabetes, Marital Status, PM_2.5_, Smoking
	13-Day	9.25	−0.22	0.06	0.0002	0.38	6	Education, Age, Physical Activity, Diabetes, Marital Status, PM_2.5_, Smoking
	2-Week	9.30	−0.21	0.06	0.0003	0.37	6	Education, Age, Physical Activity, Diabetes, Marital Status, PM_2.5_, Smoking
	1-Month	8.42	−0.21	0.06	0.001	0.37	6	Education, Age, Physical Activity, Diabetes, Marital Status, PM_2.5_, Smoking
	3-Month	7.60	−0.15	0.07	0.02	0.37	7	Education, Age, Physical Activity, Diabetes, Marital Status, Smoking, PM_2.5_
	6-Month	7.89	−0.35	0.08	<0.0001	0.38	5	Education, Age, Physical Activity, Diabetes, PM_2.5_, Marital Status, Smoking, BMI
	1-Year	7.27	−0.88	0.09	<0.0001	0.39	4	Education, Age, PM_2.5_, Physical Activity, Diabetes, Marital Status, Smoking, BMI
	2-Year	8.93	−1.09	0.10	<0.0001	0.39	3	Education, Age, PM_2.5_, Physical Activity, Diabetes, Marital Status, Smoking, BMI
	3-Year	9.35	−1.12	0.10	<0.0001	0.39	3	Education, Age, PM_2.5_, Physical Activity, Diabetes, Marital Status, Smoking, BMI
PM_10_ (µg/m^3^)								
	1-Day	25.07	-	-	-	0.37	Not Entered	Education, Age, Physical Activity, Diabetes, Marital Status, Smoking
	2-Day	21.98	-	-	-	0.37	Not Entered	Education, Age, Physical Activity, Diabetes, Marital Status, Smoking
	3-Day	20.70	-	-	-	0.37	Not Entered	Education, Age, Physical Activity, Diabetes, Marital Status, Smoking
	4-Day	19.64	-	-	-	0.37	Not Entered	Education, Age, Physical Activity, Diabetes, Marital Status, Smoking
	5-Day	18.59	-	-	-	0.37	Not Entered	Education, Age, Physical Activity, Diabetes, Marital Status, Smoking
	6-Day	17.82	-	-	-	0.37	Not Entered	Education, Age, Physical Activity, Diabetes, Marital Status, Smoking
	1-Week	17.94	-	-	-	0.37	Not Entered	Education, Age, Physical Activity, Diabetes, Marital Status, Smoking
	8-Day	18.30	-	-	-	0.37	Not Entered	Education, Age, Physical Activity, Diabetes, Marital Status, Smoking
	9-Day	18.37	-	-	-	0.37	Not Entered	Education, Age, Physical Activity, Diabetes, Marital Status, Smoking
	10-Day	17.57	-	-	-	0.37	Not Entered	Education, Age, Physical Activity, Diabetes, Marital Status, Smoking
	11-Day	17.50	-	-	-	0.37	Not Entered	Education, Age, Physical Activity, Diabetes, Marital Status, Smoking
	12-Day	17.92	-	-	-	0.37	Not Entered	Education, Age, Physical Activity, Diabetes, Marital Status, Smoking
	13-Day	18.01	-	-	-	0.37	Not Entered	Education, Age, Physical Activity, Diabetes, Marital Status, Smoking
	2-Week	18.22	-	-	-	0.37	Not Entered	Education, Age, Physical Activity, Diabetes, Marital Status, Smoking
	1-Month	18.49	-	-	-	0.37	Not Entered	Education, Age, Physical Activity, Diabetes, Marital Status, Smoking
	3-Month	15.95	0.27	0.08	0.001	0.37	6	Education, Age, Physical Activity, Diabetes, Marital Status, PM_10_, Smoking
	6-Month	9.31	0.18	0.07	0.01	0.37	7	Education, Age, Physical Activity, Diabetes, Marital Status, Smoking, PM_10_
	1-Year	4.69	−0.73	0.09	<0.0001	0.38	3	Education, Age, PM_10_, Physical Activity, Diabetes, Marital Status, Smoking, BMI
	2-Year	6.07	−0.93	0.10	<0.0001	0.39	3	Education, Age, PM_10_, Physical Activity, Diabetes, Marital Status, Smoking, BMI
	3-Year	7.15	−1.04	0.10	<0.0001	0.39	3	Education, Age, PM_10_, Physical Activity, Diabetes, Marital Status, Smoking, BMI

^a^ Effect sizes and standard errors are shown per interquartile range increase for each PM level.

## Data Availability

The data used in this study belong to the Korea National Institute of Health and the Korea Centers for Disease Control and Prevention. Due to restrictions placed on the data by the Personal Information Protection Act of Korea, the dataset cannot be made publicly available.

## Supplementary Materials

The following supporting information can be downloaded at: https://www.mdpi.com/article/10.3390/ijerph19169913/s1, Supplementary Table S1: Relationship between PM_2.5_ and PM_10_ with Decreased Cognitive Function (n = 4175). Supplementary Table S2: Relationship between Tertile of PM_2.5_ and PM_10_ and Cognitive Score (n = 4175). Supplementary Table S3: Association between PM_2.5_ and PM_10_ and Cognitive Score by Sex (Women = 2305; Men = 1870). Supplementary Table S4: Association between PM_2.5_ and PM_10_ and Cognitive Score by Age Group (50–65 years = 1665; 65–75 years = 1483; >75 years = 1027). Supplementary Table S5: Association between PM_2.5_ and PM_10_ and Cognitive Score by Alcohol Consumption (Never = 2356; Former = 318; Current = 1501). Supplementary Table S6: Association between PM_2.5_ and PM_10_ and Cognitive Score by Physical Activity (No = 2982; Yes = 1193). Supplementary Table S7: Association between PM_2.5_ and PM_10_ and Cognitive Score by Smoking Status (Never = 2750; Former = 1007; Current = 418).
