# Supplementary material for: Effect of Short- to Long-Term Exposure to Ambient Particulate Matter on Cognitive Function in a Cohort of Middle-Aged and Older Adults: KoGES
Source: Int J Environ Res Public Health. 2022 Aug 11;19(16):9913. doi: 10.3390/ijerph19169913 (PMC9408640; doi:10.3390/ijerph19169913)
Supplement: Supplementary file 1 [file ijerph-19-09913-s001.zip › ijerph-1826457-supplementary.pdf]

Supplemental Table S1. Relationship between $PM_{2.5}$ and PM_{10} with Decreased Cognitive Function (n = 4175).

Average Variables	Group	$PM_{2.5}$ ($\mu g/m^3$)				PM_{10} ($\mu g/m^3$)			
		Odds Ratio[a]	95% Confidence Interval		P-Value	Odds Ratio[a]	95% Confidence Interval		P-Value
1-Day	Total	1.00	1.00	1.01	0.63	1.00	0.99	1.00	0.15
	Tertile 2 vs. 1	1.08	0.86	1.36	0.49	0.97	0.78	1.22	0.81
	Tertile 3 vs. 1	1.14	0.91	1.44	0.25	0.87	0.69	1.11	0.26
2-Day	Total	1.01	1.00	1.01	0.09	1.00	0.99	1.00	0.61
	Tertile 2 vs. 1	1.19	0.95	1.50	0.13	0.84	0.67	1.06	0.14
	Tertile 3 vs. 1	1.29	1.03	1.63	0.03	0.92	0.72	1.18	0.52
3-Day	Total	1.01	1.00	1.02	0.01	1.00	1.00	1.01	0.58
	Tertile 2 vs. 1	1.24	0.98	1.57	0.07	0.89	0.70	1.12	0.33
	Tertile 3 vs. 1	1.32	1.04	1.68	0.02	1.00	0.78	1.28	0.98
4-Day	Total	1.02	1.01	1.03	0.001	1.00	1.00	1.01	0.23
	Tertile 2 vs. 1	1.36	1.07	1.72	0.01	1.09	0.86	1.39	0.47
	Tertile 3 vs. 1	1.53	1.20	1.94	0.001	1.11	0.85	1.44	0.45
5-Day	Total	1.02	1.01	1.03	0.001	1.00	1.00	1.01	0.24
	Tertile 2 vs. 1	1.17	0.92	1.48	0.19	1.21	0.95	1.53	0.12
	Tertile 3 vs. 1	1.52	1.20	1.94	0.001	1.25	0.94	1.65	0.13
6-Day	Total	1.02	1.01	1.03	0.001	1.01	1.00	1.01	0.28
	Tertile 2 vs. 1	1.17	0.92	1.48	0.20	1.06	0.83	1.35	0.65
	Tertile 3 vs. 1	1.45	1.14	1.86	0.003	1.22	0.91	1.64	0.18
						1.01	1.00	1.01	0.27
1-Week	Total	1.02	1.01	1.04	0.0004	1.01	1.00	1.01	0.27
	Tertile 2 vs. 1	1.24	0.98	1.57	0.08	1.13	0.89	1.44	0.32

Average Variables	Group	PM2.5 ($\mu g/m^3$)				PM10 ($\mu g/m^3$)			
		Odds Ratio[a]	95% Confidence Interval		P-Value	Odds Ratio[a]	95% Confidence Interval		P-Value
	Tertile 3 vs. 1	1.45	1.13	1.87	0.003	1.29	0.96	1.74	0.10
8-Day	Total	1.03	1.01	1.04	0.0003	1.01	1.00	1.02	0.29
	Tertile 2 vs. 1	1.39	1.09	1.77	0.01	1.08	0.85	1.37	0.55
	Tertile 3 vs. 1	1.57	1.21	2.03	0.001	1.21	0.89	1.64	0.23
9-Day	Total	1.03	1.02	1.05	0.0001	1.01	1.00	1.02	0.29
	Tertile 2 vs. 1	1.36	1.07	1.73	0.01	1.05	0.83	1.33	0.69
	Tertile 3 vs. 1	1.55	1.20	2.01	0.001	1.24	0.92	1.68	0.16
10-Day	Total	1.03	1.01	1.05	0.0002	1.00	0.99	1.01	0.52
	Tertile 2 vs. 1	1.34	1.05	1.70	0.02	1.19	0.94	1.52	0.15
	Tertile 3 vs. 1	1.54	1.19	2.00	0.001	1.30	0.96	1.77	0.10
11-Day	Total	1.03	1.02	1.05	<.0001	1.00	0.99	1.02	0.41
	Tertile 2 vs. 1	1.44	1.13	1.83	0.004	1.08	0.85	1.38	0.51
	Tertile 3 vs. 1	1.68	1.29	2.18	0.0001	1.26	0.93	1.70	0.14
12-Day	Total	1.04	1.02	1.05	<.0001	1.01	1.00	1.02	0.22
	Tertile 2 vs. 1	1.31	1.02	1.67	0.03	1.12	0.88	1.43	0.37
	Tertile 3 vs. 1	1.82	1.39	2.37	<.0001	1.24	0.91	1.68	0.17
13-Day	Total	1.04	1.02	1.05	<.0001	1.01	1.00	1.02	0.14
	Tertile 2 vs. 1	1.25	0.98	1.59	0.08	1.13	0.89	1.45	0.31
	Tertile 3 vs. 1	1.91	1.46	2.49	<.0001	1.27	0.94	1.71	0.13
2-Week	Total	1.04	1.02	1.05	<.0001	1.01	1.00	1.02	0.14
	Tertile 2 vs. 1	1.19	0.93	1.52	0.16	1.11	0.87	1.42	0.39
	Tertile 3 vs. 1	1.56	1.20	2.03	0.001	1.38	1.01	1.87	0.04

Average Variables	Group	$PM_{2.5}$ ($\mu g/m^3$)				PM_{10} ($\mu g/m^3$)			
		Odds Ratio[a]	95% Confidence Interval		P-Value	Odds Ratio[a]	95% Confidence Interval		P-Value
1-Month	Total	1.07	1.05	1.09	<.0001	1.02	1.01	1.04	0.01
	Tertile 2 vs. 1	1.64	1.27	2.13	0.0002	1.11	0.87	1.41	0.42
	Tertile 3 vs. 1	2.44	1.81	3.30	<.0001	1.52	1.07	2.15	0.02
3-Month	Total	1.13	1.09	1.17	<.0001	1.04	1.02	1.07	0.001
	Tertile 2 vs. 1	2.09	1.54	2.83	<.0001	1.74	1.24	2.43	0.001
	Tertile 3 vs. 1	3.28	2.26	4.75	<.0001	2.83	1.73	4.65	<.0001
6-Month	Total	1.18	1.13	1.23	<.0001	1.04	1.00	1.07	0.047
	Tertile 2 vs. 1	3.23	2.34	4.47	<.0001	1.31	0.87	1.96	0.19
	Tertile 3 vs. 1	3.99	2.66	6.00	<.0001	1.15	0.71	1.87	0.57
1-Year	Total	1.22	1.14	1.30	<.0001	1.10	1.04	1.17	0.002
	Tertile 2 vs. 1	4.66	2.77	7.82	<.0001	1.63	1.14	2.34	0.01
	Tertile 3 vs. 1	4.88	2.84	8.37	<.0001	1.80	1.18	2.75	0.01
2-Year	Total	1.25	1.17	1.34	<.0001	1.14	1.07	1.22	0.0001
	Tertile 2 vs. 1	3.37	1.99	5.72	<.0001	3.69	2.30	5.91	<.0001
	Tertile 3 vs. 1	3.51	1.98	6.22	<.0001	4.07	2.37	6.98	<.0001
3-Year	Total	1.20	1.11	1.30	<.0001	1.14	1.06	1.22	0.0002
	Tertile 2 vs. 1	2.24	1.28	3.92	0.01	2.78	1.67	4.63	<.0001
	Tertile 3 vs. 1	2.53	1.39	4.60	0.002	3.49	1.92	6.36	<.0001

[a] Odds ratios are shown per one-unit increase in each PM level or comparison to tertile 1 (lowest level of the PM exposure) as a reference.

Supplemental Table S2. Relationship between Tertile of $PM_{2.5}$ and PM_{10} and Cognitive Score (n = 4175).

Average Value	Group	Effect Size[a]	Standard Error[a]	P-Value	95% Confidence Interval	
$PM_{2.5}$ (μg/m³)						
1-Day	Tertile 2 vs. 1	-0.15	0.11	0.17	-0.36	0.06
	Tertile 3 vs. 1	-0.17	0.11	0.12	-0.39	0.05
2-Day	Tertile 2 vs. 1	-0.25	0.11	0.02	-0.46	-0.03
	Tertile 3 vs. 1	-0.24	0.11	0.03	-0.46	-0.03
3-Day	Tertile 2 vs. 1	-0.21	0.11	0.052	-0.43	0.002
	Tertile 3 vs. 1	-0.24	0.11	0.03	-0.46	-0.02
4-Day	Tertile 2 vs. 1	-0.31	0.11	0.006	-0.52	-0.09
	Tertile 3 vs. 1	-0.43	0.11	0.0002	-0.65	-0.20
5-Day	Tertile 2 vs. 1	-0.18	0.11	0.11	-0.39	0.04
	Tertile 3 vs. 1	-0.46	0.12	<.0001	-0.69	-0.23
6-Day	Tertile 2 vs. 1	-0.15	0.11	0.17	-0.37	0.06
	Tertile 3 vs. 1	-0.40	0.12	0.0006	-0.63	-0.17
1-Week	Tertile 2 vs. 1	-0.20	0.11	0.06	-0.42	0.01
	Tertile 3 vs. 1	-0.39	0.12	0.001	-0.62	-0.16
8-Day	Tertile 2 vs. 1	-0.49	0.11	<.0001	-0.70	-0.27
	Tertile 3 vs. 1	-0.51	0.12	<.0001	-0.74	-0.27
9-Day	Tertile 2 vs. 1	-0.39	0.11	0.0004	-0.61	-0.18
	Tertile 3 vs. 1	-0.47	0.12	0.0001	-0.71	-0.23
10-Day	Tertile 2 vs. 1	-0.42	0.11	0.0001	-0.64	-0.20
	Tertile 3 vs. 1	-0.51	0.12	<.0001	-0.75	-0.27
11-Day	Tertile 2 vs. 1	-0.45	0.11	<.0001	-0.67	-0.23
	Tertile 3 vs. 1	-0.53	0.12	<.0001	-0.77	-0.29
12-Day	Tertile 2 vs. 1	-0.34	0.11	0.002	-0.56	-0.12
	Tertile 3 vs. 1	-0.59	0.13	<.0001	-0.83	-0.34
13-Day	Tertile 2 vs. 1	-0.31	0.11	0.01	-0.53	-0.09
	Tertile 3 vs. 1	-0.64	0.12	<.0001	-0.88	-0.40
2-Week	Tertile 2 vs. 1	-0.24	0.11	0.03	-0.46	-0.02
	Tertile 3 vs. 1	-0.50	0.12	<.0001	-0.75	-0.26
1-Month	Tertile 2 vs. 1	-0.44	0.12	0.0001	-0.67	-0.21
	Tertile 3 vs. 1	-0.76	0.14	<.0001	-1.04	-0.49

Average Value	Group	Effect Size[a]	Standard Error[a]	P-Value	95% Confidence Interval	
3-Month	Tertile 2 vs. 1	-0.64	0.13	<.0001	-0.90	-0.38
	Tertile 3 vs. 1	-0.94	0.17	<.0001	-1.26	-0.61
6-Month	Tertile 2 vs. 1	-0.79	0.13	<.0001	-1.04	-0.54
	Tertile 3 vs. 1	-0.95	0.16	<.0001	-1.27	-0.64
1-Year	Tertile 2 vs. 1	-0.72	0.18	<.0001	-1.07	-0.38
	Tertile 3 vs. 1	-0.77	0.19	<.0001	-1.15	-0.40
2-Year	Tertile 2 vs. 1	-0.57	0.19	0.002	-0.94	-0.21
	Tertile 3 vs. 1	-0.76	0.21	0.0003	-1.17	-0.35
3-Year	Tertile 2 vs. 1	-0.52	0.19	0.01	-0.89	-0.15
	Tertile 3 vs. 1	-0.75	0.21	0.0004	-1.16	-0.33
PM_{10} ($\mu g/m^3$)						
1-Day	Tertile 2 vs. 1	-0.04	0.11	0.69	-0.26	0.17
	Tertile 3 vs. 1	0.11	0.12	0.35	-0.12	0.33
2-Day	Tertile 2 vs. 1	0.12	0.11	0.30	-0.10	0.33
	Tertile 3 vs. 1	0.06	0.12	0.63	-0.17	0.29
3-Day	Tertile 2 vs. 1	0.02	0.11	0.89	-0.21	0.24
	Tertile 3 vs. 1	-0.07	0.12	0.55	-0.31	0.16
4-Day	Tertile 2 vs. 1	-0.06	0.11	0.59	-0.28	0.16
	Tertile 3 vs. 1	-0.08	0.13	0.53	-0.33	0.17
5-Day	Tertile 2 vs. 1	-0.05	0.11	0.64	-0.28	0.17
	Tertile 3 vs. 1	-0.12	0.13	0.39	-0.38	0.15
6-Day	Tertile 2 vs. 1	-0.14	0.11	0.23	-0.36	0.09
	Tertile 3 vs. 1	-0.21	0.14	0.14	-0.48	0.07
1-Week	Tertile 2 vs. 1	-0.13	0.11	0.26	-0.35	0.09
	Tertile 3 vs. 1	-0.29	0.14	0.04	-0.57	-0.01
8-Day	Tertile 2 vs. 1	-0.13	0.11	0.23	-0.36	0.09
	Tertile 3 vs. 1	-0.24	0.15	0.09	-0.53	0.04
9-Day	Tertile 2 vs. 1	-0.13	0.11	0.24	-0.35	0.09
	Tertile 3 vs. 1	-0.38	0.15	0.01	-0.67	-0.10
10-Day	Tertile 2 vs. 1	-0.15	0.11	0.20	-0.37	0.08
	Tertile 3 vs. 1	-0.33	0.15	0.02	-0.62	-0.04
11-Day	Tertile 2 vs. 1	-0.09	0.12	0.46	-0.31	0.14
	Tertile 3 vs. 1	-0.30	0.15	0.04	-0.59	-0.01
12-Day	Tertile 2 vs. 1	-0.15	0.12	0.20	-0.38	0.08
	Tertile 3 vs. 1	-0.33	0.15	0.03	-0.62	-0.04

Average Value	Group	Effect Size[a]	Standard Error[a]	P-Value	95% Confidence Interval	
13-Day	Tertile 2 vs. 1	-0.17	0.12	0.14	-0.40	0.06
	Tertile 3 vs. 1	-0.28	0.15	0.06	-0.57	0.01
2-Week	Tertile 2 vs. 1	-0.21	0.12	0.07	-0.43	0.02
	Tertile 3 vs. 1	-0.37	0.15	0.01	-0.67	-0.08
1-Month	Tertile 2 vs. 1	-0.19	0.12	0.10	-0.42	0.04
	Tertile 3 vs. 1	-0.48	0.17	0.005	-0.81	-0.15
3-Month	Tertile 2 vs. 1	-0.56	0.16	0.001	-0.88	-0.25
	Tertile 3 vs. 1	-0.85	0.22	0.0001	-1.27	-0.42
6-Month	Tertile 2 vs. 1	-0.25	0.19	0.21	-0.63	0.14
	Tertile 3 vs. 1	-0.27	0.23	0.23	-0.73	0.18
1-Year	Tertile 2 vs. 1	-0.36	0.14	0.01	-0.64	-0.09
	Tertile 3 vs. 1	-0.71	0.18	<.0001	-1.05	-0.36
2-Year	Tertile 2 vs. 1	-0.65	0.16	<.0001	-0.98	-0.33
	Tertile 3 vs. 1	-1.00	0.20	<.0001	-1.40	-0.60
3-Year	Tertile 2 vs. 1	-0.62	0.17	0.0002	-0.95	-0.29
	Tertile 3 vs. 1	-1.03	0.21	<.0001	-1.45	-0.61

[a] Effect sizes and standard errors are shown per comparison to tertile 1 (lowest level of PM exposure) as a reference.

Supplemental Table S3. Association between $PM_{2.5}$ and PM_{10} and Cognitive Score by Sex (Women = 2305; Men = 1870).

Average Variables	Sex Subgroup	Estimate[a]	Standard Error[a]	P-Value	95% Confidence Limits		P for Interaction
$PM_{2.5}$							
1-Day	Men	-0.005	0.005	0.32	-0.01	0.004	0.71
	Women	-0.002	0.01	0.72	-0.01	0.01	
2-Day	Men	-0.01	0.01	0.15	-0.02	0.003	0.59
	Women	-0.01	0.01	0.28	-0.02	0.005	
3-Day	Men	-0.01	0.01	0.08	-0.02	0.001	0.32
	Women	-0.01	0.01	0.04	-0.03	-0.001	
4-Day	Men	-0.01	0.01	0.10	-0.02	0.002	0.12
	Women	-0.02	0.01	0.01	-0.03	-0.01	
5-Day	Men	-0.01	0.01	0.20	-0.02	0.005	0.04
	Women	-0.03	0.01	0.001	-0.04	-0.01	
6-Day	Men	-0.01	0.01	0.28	-0.02	0.01	0.03
	Women	-0.03	0.01	0.001	-0.04	-0.01	
1-Week	Men	-0.01	0.01	0.22	-0.03	0.01	0.03
	Women	-0.03	0.01	0.001	-0.05	-0.01	
8-Day	Men	-0.01	0.01	0.21	-0.03	0.01	0.02
	Women	-0.04	0.01	0.0002	-0.05	-0.02	
9-Day	Men	-0.01	0.01	0.23	-0.03	0.01	0.01
	Women	-0.04	0.01	<.0001	-0.06	-0.02	
10-Day	Men	-0.01	0.01	0.19	-0.03	0.01	0.01
	Women	-0.04	0.01	<.0001	-0.06	-0.02	
11-Day	Men	-0.02	0.01	0.06	-0.04	0.001	0.02
	Women	-0.05	0.01	<.0001	-0.07	-0.03	
12-Day	Men	-0.02	0.01	0.03	-0.04	-0.002	0.02
	Women	-0.05	0.01	<.0001	-0.07	-0.03	
13-Day	Men	-0.02	0.01	0.03	-0.04	-0.002	0.02
	Women	-0.05	0.01	<.0001	-0.07	-0.03	
2-Week	Men	-0.02	0.01	0.05	-0.04	0.0002	0.02
	Women	-0.05	0.01	<.0001	-0.07	-0.03	
1-Month	Men	-0.03	0.01	0.03	-0.05	-0.002	0.01
	Women	-0.07	0.01	<.0001	-0.10	-0.05	
3-Month	Men	-0.05	0.02	0.01	-0.09	-0.01	0.01
	Women	-0.11	0.02	<.0001	-0.16	-0.07	
6-Month	Men	-0.05	0.02	0.03	-0.09	-0.005	0.001
	Women	-0.14	0.02	<.0001	-0.19	-0.10	
1-Year	Men	-0.04	0.03	0.12	-0.09	0.01	<.0001
	Women	-0.16	0.03	<.0001	-0.22	-0.09	
2-Year	Men	-0.08	0.03	0.01	-0.14	-0.02	<.0001
	Women	-0.18	0.04	<.0001	-0.25	-0.11	
3-Year	Men	-0.07	0.03	0.02	-0.13	-0.01	<.0001
	Women	-0.16	0.04	<.0001	-0.23	-0.09	
PM_{10}							

Average Variables	Sex Subgroup	Estimate[a]	Standard Error[a]	P-Value	95% Confidence Limits		P for Interaction
1-Day	Men	-0.001	0.003	0.66	-0.01	0.004	0.32
	Women	0.004	0.003	0.17	-0.002	0.01	
2-Day	Men	-0.003	0.003	0.43	-0.01	0.004	0.41
	Women	0.003	0.004	0.46	-0.005	0.01	
3-Day	Men	-0.003	0.004	0.36	-0.01	0.004	0.76
	Women	-0.002	0.004	0.67	-0.01	0.01	
4-Day	Men	-0.004	0.004	0.38	-0.01	0.005	0.93
	Women	-0.004	0.005	0.33	-0.01	0.004	
5-Day	Men	-0.003	0.005	0.46	-0.01	0.01	0.87
	Women	-0.01	0.01	0.20	-0.02	0.003	
6-Day	Men	-0.004	0.01	0.50	-0.01	0.01	0.91
	Women	-0.01	0.01	0.23	-0.02	0.004	
1-Week	Men	-0.004	0.01	0.50	-0.01	0.01	0.98
	Women	-0.01	0.01	0.26	-0.02	0.01	
8-Day	Men	-0.004	0.01	0.50	-0.02	0.01	0.89
	Women	-0.01	0.01	0.17	-0.02	0.004	
9-Day	Men	-0.003	0.01	0.60	-0.02	0.01	0.72
	Women	-0.01	0.01	0.13	-0.02	0.003	
10-Day	Men	-0.002	0.01	0.79	-0.01	0.01	0.62
	Women	-0.01	0.01	0.14	-0.02	0.003	
11-Day	Men	-0.01	0.01	0.43	-0.02	0.01	0.83
	Women	-0.01	0.01	0.15	-0.02	0.004	
12-Day	Men	-0.01	0.01	0.29	-0.02	0.01	0.95
	Women	-0.01	0.01	0.14	-0.02	0.003	
13-Day	Men	-0.01	0.01	0.24	-0.02	0.01	0.91
	Women	-0.01	0.01	0.08	-0.03	0.002	
2-Week	Men	-0.01	0.01	0.33	-0.02	0.01	0.78
	Women	-0.01	0.01	0.05	-0.03	-0.0001	
1-Month	Men	-0.01	0.01	0.16	-0.03	0.01	0.89
	Women	-0.03	0.01	0.02	-0.05	-0.005	
3-Month	Men	-0.03	0.01	0.04	-0.06	-0.001	0.61
	Women	-0.05	0.02	0.01	-0.08	-0.01	
6-Month	Men	-0.03	0.02	0.19	-0.06	0.01	0.90
	Women	-0.06	0.02	0.01	-0.11	-0.02	
1-Year	Men	-0.05	0.03	0.10	-0.12	0.01	<.0001
	Women	-0.12	0.04	0.003	-0.20	-0.04	
2-Year	Men	-0.08	0.03	0.01	-0.15	-0.02	<.0001
	Women	-0.15	0.04	0.0002	-0.23	-0.07	
3-Year	Men	-0.08	0.03	0.01	-0.14	-0.02	<.0001
	Women	-0.15	0.04	0.0002	-0.22	-0.07	

[a] Effect sizes and standard errors are shown per each unit increase in cognitive score.

Supplemental Table S4. Association between $PM_{2.5}$ and PM_{10} and Cognitive Score by Age Group (50-65 yrs = 1665; 65-75 yrs = 1483; > 75 yrs = 1027).

Average Variables	Age Subgroup	Effect Size[a]	Standard Error[a]	P-Value	95% Confidence Limits		P for Interaction
$PM_{2.5}$							
1-Day	50-65	-0.01	0.004	0.001	-0.02	-0.01	0.18
	65-75	-0.003	0.01	0.56	-0.01	0.01	
	>75	0.001	0.01	0.89	-0.02	0.02	
2-Day	50-65	-0.01	0.005	0.002	-0.02	-0.01	0.60
	65-75	-0.01	0.01	0.19	-0.02	0.004	
	>75	-0.01	0.01	0.56	-0.03	0.01	
3-Day	50-65	-0.02	0.01	0.003	-0.03	-0.01	0.99
	65-75	-0.02	0.01	0.04	-0.03	-0.001	
	>75	-0.02	0.01	0.14	-0.04	0.01	
4-Day	50-65	-0.02	0.01	0.001	-0.03	-0.01	0.92
	65-75	-0.02	0.01	0.02	-0.04	-0.003	
	>75	-0.03	0.01	0.06	-0.05	0.002	
5-Day	50-65	-0.02	0.01	0.0002	-0.03	-0.01	0.79
	65-75	-0.02	0.01	0.03	-0.04	-0.002	
	>75	-0.03	0.02	0.04	-0.06	-0.001	
6-Day	50-65	-0.02	0.01	0.0003	-0.04	-0.01	0.76
	65-75	-0.02	0.01	0.04	-0.04	-0.001	
	>75	-0.03	0.02	0.04	-0.07	-0.002	
1-Week	50-65	-0.02	0.01	0.001	-0.04	-0.01	0.64
	65-75	-0.03	0.01	0.01	-0.05	-0.01	
	>75	-0.04	0.02	0.03	-0.07	-0.003	
8-Day	50-65	-0.02	0.01	0.003	-0.04	-0.01	0.33
	65-75	-0.03	0.01	0.01	-0.05	-0.01	
	>75	-0.05	0.02	0.01	-0.09	-0.01	
9-Day	50-65	-0.02	0.01	0.01	-0.04	-0.01	0.19
	65-75	-0.03	0.01	0.003	-0.06	-0.01	
	>75	-0.06	0.02	0.01	-0.09	-0.02	
10-Day	50-65	-0.02	0.01	0.004	-0.04	-0.01	0.23
	65-75	-0.04	0.01	0.001	-0.06	-0.01	
	>75	-0.06	0.02	0.005	-0.09	-0.02	
11-Day	50-65	-0.03	0.01	0.001	-0.05	-0.01	0.28
	65-75	-0.04	0.01	0.0003	-0.07	-0.02	
	>75	-0.06	0.02	0.003	-0.10	-0.02	
12-Day	50-65	-0.03	0.01	0.001	-0.05	-0.01	0.29
	65-75	-0.05	0.01	0.0001	-0.07	-0.02	
	>75	-0.06	0.02	0.002	-0.10	-0.02	
13-Day	50-65	-0.03	0.01	0.0003	-0.05	-0.02	0.27
	65-75	-0.05	0.01	0.0001	-0.07	-0.02	
	>75	-0.06	0.02	0.001	-0.10	-0.03	
2-Week	50-65	-0.04	0.01	0.0001	-0.05	-0.02	0.28

Average Variables	Age Subgroup	Effect Size[a]	Standard Error[a]	P-Value	95% Confidence Limits		P for Interaction
	65-75	-0.05	0.01	0.0003	-0.07	-0.02	
	>75	-0.06	0.02	0.001	-0.10	-0.03	
1-Month	50-65	-0.06	0.01	<.0001	-0.08	-0.03	0.13
	65-75	-0.05	0.02	0.001	-0.09	-0.02	
	>75	-0.11	0.03	<.0001	-0.16	-0.06	
3-Month	50-65	-0.09	0.02	<.0001	-0.12	-0.06	0.78
	65-75	-0.11	0.02	<.0001	-0.16	-0.07	
	>75	-0.14	0.04	0.002	-0.23	-0.05	
6-Month	50-65	-0.09	0.02	<.0001	-0.12	-0.06	0.43
	65-75	-0.12	0.03	<.0001	-0.17	-0.07	
	>75	-0.24	0.05	<.0001	-0.34	-0.14	
1-Year	50-65	-0.09	0.02	<.0001	-0.13	-0.05	<.0001
	65-75	-0.12	0.04	0.001	-0.19	-0.05	
	>75	-0.27	0.08	0.001	-0.44	-0.11	
2-Year	50-65	-0.10	0.03	<.0001	-0.15	-0.05	<.0001
	65-75	-0.14	0.04	0.0002	-0.22	-0.07	
	>75	-0.39	0.08	<.0001	-0.55	-0.22	
3-Year	50-65	-0.08	0.02	0.001	-0.13	-0.04	<.0001
	65-75	-0.13	0.04	0.002	-0.21	-0.05	
	>75	-0.38	0.10	0.0001	-0.57	-0.19	
PM_{10}							
1-Day	50-65	-0.004	0.003	0.15	-0.01	0.001	0.06
	65-75	0.002	0.004	0.67	-0.01	0.01	
	>75	0.01	0.01	0.19	-0.004	0.02	
2-Day	50-65	-0.004	0.003	0.20	-0.01	0.002	0.11
	65-75	-0.003	0.004	0.511	-0.011	0.006	
	>75	0.01	0.01	0.28	-0.01	0.02	
3-Day	50-65	-0.004	0.003	0.19	-0.01	0.002	0.34
	65-75	-0.01	0.005	0.14	-0.02	0.002	
	>75	0.002	0.01	0.79	-0.01	0.02	
4-Day	50-65	-0.005	0.004	0.16	-0.01	0.002	0.68
	65-75	-0.01	0.01	0.12	-0.02	0.002	
	>75	-0.003	0.01	0.74	-0.02	0.01	
5-Day	50-65	-0.01	0.004	0.10	-0.01	0.001	0.82
	65-75	-0.01	0.01	0.20	-0.02	0.004	
	>75	-0.01	0.01	0.55	-0.03	0.01	
6-Day	50-65	-0.01	0.004	0.14	-0.01	0.002	0.86
	65-75	-0.01	0.01	0.31	-0.02	0.01	
	>75	-0.01	0.01	0.51	-0.03	0.01	
1-Week	50-65	-0.005	0.005	0.32	-0.01	0.004	0.96
	65-75	-0.01	0.01	0.35	-0.02	0.01	
	>75	-0.01	0.01	0.43	-0.03	0.01	
8-Day	50-65	-0.004	0.005	0.47	-0.01	0.01	0.99
	65-75	-0.01	0.01	0.31	-0.02	0.01	
	>75	-0.02	0.01	0.22	-0.04	0.01	

Average Variables	Age Subgroup	Effect Size[a]	Standard Error[a]	P-Value	95% Confidence Limits		P for Interaction
9-Day	50-65	-0.002	0.01	0.71	-0.01	0.01	0.94
	65-75	-0.01	0.01	0.22	-0.03	0.01	
	>75	-0.02	0.01	0.16	-0.05	0.01	
10-Day	50-65	-0.002	0.01	0.67	-0.01	0.01	0.98
	65-75	-0.01	0.01	0.26	-0.03	0.01	
	>75	-0.02	0.01	0.23	-0.04	0.01	
11-Day	50-65	-0.005	0.01	0.39	-0.02	0.01	1.00
	65-75	-0.01	0.01	0.16	-0.03	0.005	
	>75	-0.02	0.01	0.24	-0.04	0.01	
12-Day	50-65	-0.01	0.01	0.28	-0.02	0.01	0.99
	65-75	-0.01	0.01	0.11	-0.03	0.003	
	>75	-0.02	0.01	0.24	-0.04	0.01	
13-Day	50-65	-0.01	0.01	0.20	-0.02	0.004	1.00
	65-75	-0.02	0.01	0.08	-0.03	0.002	
	>75	-0.02	0.01	0.18	-0.04	0.01	
2-Week	50-65	-0.01	0.01	0.13	-0.02	0.003	0.98
	65-75	-0.01	0.01	0.09	-0.03	0.002	
	>75	-0.02	0.01	0.17	-0.04	0.01	
1-Month	50-65	-0.03	0.01	0.004	-0.04	-0.01	0.67
	65-75	-0.02	0.01	0.12	-0.05	0.01	
	>75	-0.04	0.02	0.05	-0.08	0.0005	
3-Month	50-65	-0.05	0.01	0.0003	-0.07	-0.02	0.02
	65-75	-0.06	0.02	0.002	-0.09	-0.02	
	>75	-0.03	0.03	0.33	-0.09	0.03	
6-Month	50-65	-0.06	0.02	0.002	-0.09	-0.02	0.01
	65-75	-0.06	0.03	0.02	-0.11	-0.01	
	>75	-0.05	0.04	0.24	-0.14	0.03	
1-Year	50-65	-0.12	0.03	<.0001	-0.18	-0.06	0.01
	65-75	-0.11	0.04	0.01	-0.20	-0.03	
	>75	-0.05	0.09	0.59	-0.23	0.13	
2-Year	50-65	-0.12	0.03	<.0001	-0.17	-0.06	<.0001
	65-75	-0.13	0.04	0.003	-0.21	-0.04	
	>75	-0.25	0.10	0.01	-0.44	-0.07	
3-Year	50-65	-0.11	0.03	<.0001	-0.16	-0.05	<.0001
	65-75	-0.12	0.04	0.003	-0.20	-0.04	
	>75	-0.27	0.10	0.01	-0.47	-0.07	

[a] Effect sizes and standard errors are shown per each unit increase in cognitive score.

Supplemental Table S5. Association between $PM_{2.5}$ and PM_{10} and Cognitive Score by Alcohol Consumption (Never = 2356; Former = 318; Current = 1501).

Average Variables	Alcohol Consumption Subgroup	Estimate[a]	Standard Error[a]	P-Value	95% Confidence Limits		P for Interaction
$PM_{2.5}$							
1-Day	Never	-0.001	0.01	0.84	-0.01	0.01	0.98
	Former	0.002	0.01	0.89	-0.02	0.02	
	Current	-0.01	0.01	0.11	-0.02	0.002	
2-Day	Never	-0.01	0.01	0.37	-0.02	0.01	0.97
	Former	-0.0005	0.01	0.97	-0.03	0.02	
	Current	-0.01	0.01	0.05	-0.02	-0.0001	
3-Day	Never	-0.01	0.01	0.03	-0.03	-0.001	0.74
	Former	-0.01	0.01	0.64	-0.04	0.02	
	Current	-0.01	0.01	0.07	-0.03	0.001	
4-Day	Never	-0.02	0.01	0.01	-0.03	-0.01	0.57
	Former	-0.01	0.02	0.52	-0.04	0.02	
	Current	-0.01	0.01	0.05	-0.03	-0.0001	
5-Day	Never	-0.02	0.01	0.002	-0.04	-0.01	0.33
	Former	-0.01	0.02	0.66	-0.05	0.03	
	Current	-0.02	0.01	0.07	-0.03	0.001	
6-Day	Never	-0.03	0.01	0.001	-0.04	-0.01	0.24
	Former	-0.01	0.02	0.74	-0.05	0.03	
	Current	-0.01	0.01	0.10	-0.03	0.003	
1-Week	Never	-0.03	0.01	0.001	-0.05	-0.01	0.30
	Former	-0.01	0.02	0.79	-0.05	0.04	
	Current	-0.02	0.01	0.05	-0.04	0.0004	
8-Day	Never	-0.03	0.01	0.001	-0.05	-0.01	0.38
	Former	0.001	0.03	0.98	-0.05	0.05	
	Current	-0.02	0.01	0.02	-0.04	-0.005	
9-Day	Never	-0.03	0.01	0.0005	-0.05	-0.01	0.42
	Former	0.001	0.03	0.96	-0.05	0.05	
	Current	-0.03	0.01	0.01	-0.05	-0.01	
10-Day	Never	-0.04	0.01	0.0004	-0.05	-0.02	0.48
	Former	-0.0003	0.03	0.99	-0.06	0.06	
	Current	-0.03	0.01	0.004	-0.05	-0.01	
11-Day	Never	-0.04	0.01	0.0001	-0.06	-0.02	0.53
	Former	-0.01	0.03	0.85	-0.06	0.05	
	Current	-0.04	0.01	0.001	-0.06	-0.01	
12-Day	Never	-0.04	0.01	<.0001	-0.06	-0.02	0.65

Average Variables	Alcohol Consumption Subgroup	Estimate[a]	Standard Error[a]	P-Value	95% Confidence Limits		P for Interaction
	Former	-0.003	0.03	0.93	-0.06	0.06	
	Current	-0.04	0.01	0.0004	-0.07	-0.02	
13-Day	Never	-0.04	0.01	<.0001	-0.06	-0.02	0.68
	Former	0.001	0.03	0.97	-0.06	0.06	
	Current	-0.05	0.01	0.0002	-0.07	-0.02	
2-Week	Never	-0.04	0.01	<.0001	-0.06	-0.02	0.60
	Former	0.01	0.03	0.73	-0.05	0.07	
	Current	-0.05	0.01	0.0002	-0.07	-0.02	
1-Month	Never	-0.06	0.01	<.0001	-0.09	-0.04	0.31
	Former	0.004	0.04	0.92	-0.07	0.08	
	Current	-0.06	0.02	0.0001	-0.09	-0.03	
3-Month	Never	-0.10	0.02	<.0001	-0.14	-0.06	0.33
	Former	-0.02	0.06	0.68	-0.13	0.09	
	Current	-0.09	0.02	<.0001	-0.13	-0.04	
6-Month	Never	-0.13	0.02	<.0001	-0.17	-0.08	<.0001
	Former	-0.02	0.06	0.80	-0.13	0.10	
	Current	-0.09	0.02	0.0002	-0.14	-0.04	
1-Year	Never	-0.15	0.03	<.0001	-0.21	-0.09	0.01
	Former	0.06	0.08	0.46	-0.09	0.20	
	Current	-0.09	0.03	0.004	-0.15	-0.03	
2-Year	Never	-0.19	0.03	<.0001	-0.25	-0.12	0.002
	Former	0.03	0.08	0.69	-0.13	0.20	
	Current	-0.11	0.04	0.004	-0.18	-0.03	
3-Year	Never	-0.17	0.04	<.0001	-0.24	-0.10	0.002
	Former	0.07	0.08	0.43	-0.10	0.23	
	Current	-0.11	0.04	0.004	-0.18	-0.03	
PM_{10}							
1-Day	Never	0.01	0.003	0.05	-0.0001	0.01	0.48
	Former	0.002	0.01	0.80	-0.01	0.02	
	Current	-0.005	0.003	0.13	-0.01	0.001	
2-Day	Never	0.01	0.004	0.13	-0.002	0.01	0.49
	Former	-0.001	0.01	0.89	-0.02	0.02	
	Current	-0.01	0.004	0.05	-0.01	0.0001	
3-Day	Never	0.001	0.004	0.81	-0.01	0.01	0.94
	Former	-0.01	0.01	0.47	-0.03	0.01	
	Current	-0.01	0.004	0.08	-0.02	0.001	
4-Day	Never	-0.002	0.004	0.65	-0.01	0.01	1.00
	Former	-0.01	0.01	0.43	-0.03	0.01	

Average Variables	Alcohol Consumption Subgroup	Estimate[a]	Standard Error[a]	P-Value	95% Confidence Limits		P for Interaction
	Current	-0.01	0.005	0.12	-0.02	0.002	
5-Day	Never	-0.004	0.01	0.41	-0.01	0.01	0.99
	Former	-0.01	0.01	0.51	-0.03	0.02	
	Current	-0.01	0.01	0.16	-0.02	0.003	
6-Day	Never	-0.01	0.01	0.34	-0.02	0.01	0.90
	Former	-0.01	0.01	0.46	-0.04	0.02	
	Current	-0.01	0.01	0.28	-0.02	0.01	
1-Week	Never	-0.01	0.01	0.31	-0.02	0.01	0.82
	Former	-0.01	0.02	0.42	-0.04	0.02	
	Current	-0.01	0.01	0.34	-0.02	0.01	
8-Day	Never	-0.01	0.01	0.26	-0.02	0.01	0.85
	Former	-0.01	0.02	0.55	-0.04	0.02	
	Current	-0.01	0.01	0.20	-0.02	0.005	
9-Day	Never	-0.01	0.01	0.24	-0.02	0.01	0.83
	Former	-0.01	0.02	0.63	-0.04	0.03	
	Current	-0.01	0.01	0.18	-0.02	0.004	
10-Day	Never	-0.01	0.01	0.30	-0.02	0.01	0.82
	Former	-0.01	0.02	0.70	-0.04	0.03	
	Current	-0.01	0.01	0.22	-0.02	0.01	
11-Day	Never	-0.01	0.01	0.22	-0.02	0.01	0.81
	Former	-0.01	0.02	0.64	-0.05	0.03	
	Current	-0.01	0.01	0.18	-0.03	0.005	
12-Day	Never	-0.01	0.01	0.19	-0.02	0.005	0.90
	Former	-0.01	0.02	0.77	-0.04	0.03	
	Current	-0.01	0.01	0.10	-0.03	0.003	
13-Day	Never	-0.01	0.01	0.14	-0.02	0.003	0.92
	Former	-0.003	0.02	0.88	-0.04	0.03	
	Current	-0.02	0.01	0.05	-0.03	0.000	
2-Week	Never	-0.01	0.01	0.12	-0.02	0.003	0.88
	Former	0.004	0.02	0.85	-0.03	0.04	
	Current	-0.02	0.01	0.04	-0.03	-0.001	
1-Month	Never	-0.02	0.01	0.10	-0.04	0.003	0.84
	Former	-0.01	0.03	0.72	-0.06	0.04	
	Current	-0.03	0.01	0.01	-0.05	-0.01	
3-Month	Never	-0.04	0.02	0.03	-0.07	-0.004	0.35
	Former	0.01	0.04	0.88	-0.08	0.09	
	Current	-0.06	0.02	0.001	-0.09	-0.02	
6-Month	Never	-0.05	0.02	0.02	-0.10	-0.01	0.56

Average Variables	Alcohol Consumption Subgroup	Estimate[a]	Standard Error[a]	P-Value	95% Confidence Limits		P for Interaction
	Former	0.04	0.05	0.48	-0.07	0.15	
	Current	-0.05	0.02	0.02	-0.10	-0.01	
1-Year	Never	-0.13	0.04	0.002	-0.20	-0.05	0.02
	Former	0.03	0.10	0.73	-0.16	0.22	
	Current	-0.08	0.04	0.03	-0.15	-0.01	
2-Year	Never	-0.18	0.04	<.0001	-0.26	-0.10	0.002
	Former	0.02	0.10	0.84	-0.17	0.21	
	Current	-0.08	0.04	0.03	-0.16	-0.01	
3-Year	Never	-0.16	0.04	<.0001	-0.24	-0.09	0.001
	Former	0.04	0.09	0.67	-0.14	0.21	
	Current	-0.09	0.04	0.01	-0.16	-0.02	

[a] Effect sizes and standard errors are shown per each unit increase in cognitive score.

Supplemental Table S6. Association between $PM_{2.5}$ and PM_{10} and Cognitive Score by Physical Activity (No = 2982; Yes = 1193).

Average Variables	Physical Activity Subgroup	Estimate[a]	Standard Error[a]	P-Value	95% Confidence Limits		P for Interaction
$PM_{2.5}$							
1-Day	No	-0.001	0.004	0.78	-0.01	0.01	0.69
	Yes	-0.01	0.01	0.13	-0.02	0.003	
2-Day	No	-0.004	0.005	0.35	-0.01	0.005	0.71
	Yes	-0.02	0.01	0.01	-0.03	-0.003	
3-Day	No	-0.01	0.01	0.04	-0.02	-0.001	0.69
	Yes	-0.02	0.01	0.02	-0.03	-0.003	
4-Day	No	-0.02	0.01	0.01	-0.03	-0.003	0.64
	Yes	-0.02	0.01	0.01	-0.03	-0.005	
5-Day	No	-0.02	0.01	0.01	-0.03	-0.005	0.56
	Yes	-0.02	0.01	0.01	-0.04	-0.01	
6-Day	No	-0.02	0.01	0.01	-0.03	-0.01	0.37
	Yes	-0.02	0.01	0.03	-0.04	-0.002	
1-Week	No	-0.02	0.01	0.004	-0.04	-0.01	0.41
	Yes	-0.02	0.01	0.02	-0.04	-0.003	
8-Day	No	-0.03	0.01	0.002	-0.04	-0.01	0.32
	Yes	-0.02	0.01	0.03	-0.04	-0.002	
9-Day	No	-0.03	0.01	0.001	-0.05	-0.01	0.20
	Yes	-0.02	0.01	0.06	-0.04	0.001	
10-Day	No	-0.03	0.01	0.0002	-0.05	-0.02	0.11
	Yes	-0.02	0.01	0.09	-0.04	0.003	
11-Day	No	-0.04	0.01	<.0001	-0.06	-0.02	0.09
	Yes	-0.02	0.01	0.07	-0.04	0.002	
12-Day	No	-0.04	0.01	<.0001	-0.06	-0.02	0.08
	Yes	-0.02	0.01	0.05	-0.04	0.0001	
13-Day	No	-0.04	0.01	<.0001	-0.06	-0.03	0.08
	Yes	-0.02	0.01	0.05	-0.04	0.00001	
2-Week	No	-0.04	0.01	<.0001	-0.06	-0.03	0.07
	Yes	-0.02	0.01	0.06	-0.04	0.001	
1-Month	No	-0.06	0.01	<.0001	-0.08	-0.04	0.07
	Yes	-0.04	0.01	0.0016	-0.07	-0.02	
3-Month	No	-0.09	0.02	<.0001	-0.13	-0.06	0.03
	Yes	-0.08	0.02	<.0001	-0.12	-0.04	
6-Month	No	-0.12	0.02	<.0001	-0.16	-0.08	0.001
	Yes	-0.08	0.02	0.0001	-0.12	-0.04	

Average Variables	Physical Activity Subgroup	Estimate[a]	Standard Error[a]	P-Value	95% Confidence Limits		P for Interaction
1-Year	No	-0.13	0.03	<.0001	-0.19	-0.07	<.0001
	Yes	-0.07	0.03	0.01	-0.12	-0.02	
2-Year	No	-0.18	0.03	<.0001	-0.24	-0.11	<.0001
	Yes	-0.07	0.03	0.02	-0.13	-0.01	
3-Year	No	-0.17	0.04	<.0001	-0.24	-0.10	<.0001
	Yes	-0.06	0.03	0.03	-0.12	-0.004	
PM_{10}							
1-Day	No	0.003	0.003	0.23	-0.002	0.01	0.45
	Yes	-0.002	0.003	0.64	-0.01	0.01	
2-Day	No	0.002	0.003	0.54	-0.004	0.01	0.48
	Yes	-0.004	0.004	0.28	-0.01	0.003	
3-Day	No	-0.002	0.004	0.55	-0.01	0.005	0.94
	Yes	-0.004	0.004	0.37	-0.01	0.004	
4-Day	No	-0.004	0.004	0.37	-0.01	0.004	1.00
	Yes	-0.01	0.005	0.21	-0.02	0.003	
5-Day	No	-0.01	0.004	0.26	-0.01	0.004	0.86
	Yes	-0.01	0.01	0.25	-0.02	0.004	
6-Day	No	-0.01	0.01	0.24	-0.02	0.004	0.65
	Yes	-0.004	0.01	0.52	-0.02	0.01	
1-Week	No	-0.01	0.01	0.31	-0.02	0.01	0.76
	Yes	-0.005	0.01	0.47	-0.02	0.01	
8-Day	No	-0.01	0.01	0.21	-0.02	0.004	0.64
	Yes	-0.004	0.01	0.57	-0.02	0.01	
9-Day	No	-0.01	0.01	0.16	-0.02	0.003	0.47
	Yes	-0.002	0.01	0.80	-0.02	0.01	
10-Day	No	-0.01	0.01	0.18	-0.02	0.004	0.38
	Yes	0.0003	0.01	0.96	-0.01	0.01	
11-Day	No	-0.01	0.01	0.12	-0.02	0.003	0.34
	Yes	-0.0002	0.01	0.98	-0.01	0.01	
12-Day	No	-0.01	0.01	0.09	-0.02	0.002	0.36
	Yes	-0.002	0.01	0.78	-0.02	0.01	
13-Day	No	-0.01	0.01	0.05	-0.03	-0.0002	0.35
	Yes	-0.003	0.01	0.71	-0.02	0.01	
2-Week	No	-0.01	0.01	0.03	-0.03	-0.001	0.32
	Yes	-0.002	0.01	0.75	-0.02	0.01	
1-Month	No	-0.02	0.01	0.03	-0.04	-0.002	0.56
	Yes	-0.02	0.01	0.07	-0.04	0.002	

Average Variables	Physical Activity Subgroup	Estimate[a]	Standard Error[a]	P-Value	95% Confidence Limits		P for Interaction
3-Month	No	-0.04	0.01	0.01	-0.07	-0.01	0.61
	Yes	-0.04	0.02	0.01	-0.08	-0.01	
6-Month	No	-0.05	0.02	0.01	-0.09	-0.01	0.20
	Yes	-0.04	0.02	0.05	-0.09	0.00004	
1-Year	No	-0.10	0.04	0.004	-0.17	-0.03	<.0001
	Yes	-0.08	0.04	0.03	-0.15	-0.01	
2-Year	No	-0.16	0.04	<.0001	-0.23	-0.08	<.0001
	Yes	-0.07	0.03	0.03	-0.14	-0.01	
3-Year	No	-0.15	0.04	<.0001	-0.22	-0.08	<.0001
	Yes	-0.07	0.03	0.02	-0.12	-0.01	

[a] Effect sizes and standard errors are shown per each unit increase in cognitive score.

Supplemental Table S7. Association between $PM_{2.5}$ and PM_{10} and Cognitive Score by Smoking Status (Never = 2750; Former = 1007; Current = 418).

Average Variables	Smoking Subgroup	Estimate[a]	Standard Error[a]	P-Value	95% Confidence Limits		P for Interaction
$PM_{2.5}$							
1-Day	Never	-0.003	0.005	0.54	-0.01	0.01	0.11
	Former	0.01	0.01	0.40	-0.01	0.02	
	Current	-0.03	0.01	0.01	-0.05	-0.01	
2-Day	Never	-0.01	0.01	0.19	-0.02	0.003	0.16
	Former	0.002	0.01	0.81	-0.01	0.02	
	Current	-0.03	0.01	0.005	-0.06	-0.01	
3-Day	Never	-0.01	0.01	0.02	-0.02	-0.002	0.28
	Former	-0.002	0.01	0.84	-0.02	0.01	
	Current	-0.04	0.01	0.01	-0.07	-0.01	
4-Day	Never	-0.02	0.01	0.01	-0.03	-0.01	0.13
	Former	-0.005	0.01	0.58	-0.02	0.01	
	Current	-0.04	0.02	0.01	-0.07	-0.01	
5-Day	Never	-0.02	0.01	0.002	-0.03	-0.01	0.47
	Former	-0.01	0.01	0.57	-0.02	0.01	
	Current	-0.04	0.02	0.02	-0.07	-0.005	
6-Day	Never	-0.02	0.01	0.002	-0.04	-0.01	0.48
	Former	-0.01	0.01	0.57	-0.03	0.01	
	Current	-0.03	0.02	0.07	-0.07	0.003	
1-Week	Never	-0.03	0.01	0.001	-0.04	-0.01	0.40
	Former	-0.01	0.01	0.56	-0.03	0.02	
	Current	-0.03	0.02	0.11	-0.07	0.01	
8-Day	Never	-0.03	0.01	0.0001	-0.05	-0.02	0.25
	Former	-0.01	0.01	0.62	-0.03	0.02	
	Current	-0.03	0.02	0.20	-0.07	0.01	
9-Day	Never	-0.04	0.01	<.0001	-0.06	-0.02	0.11
	Former	-0.002	0.01	0.85	-0.03	0.02	
	Current	-0.02	0.02	0.25	-0.07	0.02	
10-Day	Never	-0.04	0.01	<.0001	-0.06	-0.02	0.08
	Former	-0.003	0.01	0.85	-0.03	0.02	
	Current	-0.02	0.02	0.29	-0.07	0.02	
11-Day	Never	-0.05	0.01	<.0001	-0.06	-0.03	0.09
	Former	-0.01	0.01	0.68	-0.03	0.02	
	Current	-0.03	0.02	0.17	-0.08	0.01	
12-Day	Never	-0.05	0.01	<.0001	-0.07	-0.03	0.11
	Former	-0.01	0.01	0.51	-0.04	0.02	
	Current	-0.04	0.02	0.12	-0.08	0.01	
13-Day	Never	-0.05	0.01	<.0001	-0.07	-0.03	0.10

Average Variables	Smoking Subgroup	Estimate[a]	Standard Error[a]	P-Value	95% Confidence Limits		P for Interaction
	Former	-0.01	0.01	0.57	-0.04	0.02	
	Current	-0.04	0.02	0.06	-0.09	0.002	
2-Week	Never	-0.05	0.01	<.0001	-0.07	-0.03	0.06
	Former	-0.004	0.01	0.76	-0.03	0.02	
	Current	-0.04	0.02	0.08	-0.09	0.005	
1-Month	Never	-0.07	0.01	<.0001	-0.10	-0.05	0.04
	Former	-0.001	0.02	0.93	-0.04	0.03	
	Current	-0.04	0.03	0.22	-0.10	0.02	
3-Month	Never	-0.11	0.02	<.0001	-0.15	-0.07	0.26
	Former	-0.04	0.03	0.11	-0.09	0.01	
	Current	-0.03	0.05	0.56	-0.13	0.07	
6-Month	Never	-0.14	0.02	<.0001	-0.18	-0.10	0.02
	Former	-0.04	0.03	0.13	-0.09	0.01	
	Current	0.01	0.05	0.80	-0.09	0.11	
1-Year	Never	-0.15	0.03	<.0001	-0.20	-0.09	<.0001
	Former	-0.04	0.03	0.26	-0.11	0.03	
	Current	-0.04	0.07	0.56	-0.17	0.09	
2-Year	Never	-0.18	0.03	<.0001	-0.24	-0.12	<.0001
	Former	-0.04	0.04	0.30	-0.12	0.04	
	Current	-0.09	0.07	0.22	-0.23	0.05	
3-Year	Never	-0.16	0.03	<.0001	-0.23	-0.10	<.0001
	Former	-0.03	0.04	0.50	-0.11	0.05	
	Current	-0.09	0.07	0.20	-0.22	0.05	
$PM1_0$							
1-Day	Never	0.003	0.003	0.29	-0.003	0.01	0.09
	Former	0.005	0.004	0.22	-0.003	0.01	
	Current	-0.02	0.01	0.01	-0.03	-0.01	
2-Day	Never	0.001	0.003	0.73	-0.01	0.01	0.12
	Former	0.005	0.004	0.30	-0.004	0.01	
	Current	-0.02	0.01	0.005	-0.04	-0.01	
3-Day	Never	-0.003	0.004	0.42	-0.01	0.004	0.22
	Former	0.005	0.01	0.37	-0.01	0.01	
	Current	-0.02	0.01	0.01	-0.04	-0.01	
4-Day	Never	-0.005	0.004	0.24	-0.01	0.003	0.47
	Former	0.003	0.01	0.59	-0.01	0.01	
	Current	-0.02	0.01	0.02	-0.04	-0.004	
5-Day	Never	-0.01	0.005	0.20	-0.01	0.003	0.69
	Former	0.002	0.01	0.76	-0.01	0.01	
	Current	-0.02	0.01	0.04	-0.04	-0.001	
6-Day	Never	-0.01	0.01	0.21	-0.02	0.004	0.81
	Former	0.002	0.01	0.81	-0.01	0.02	
	Current	-0.02	0.01	0.09	-0.04	0.003	

Average Variables	Smoking Subgroup	Estimate[a]	Standard Error[a]	P-Value	95% Confidence Limits		P for Interaction
1-Week	Never	-0.01	0.01	0.19	-0.02	0.004	0.89
	Former	0.003	0.01	0.72	-0.01	0.02	
	Current	-0.02	0.01	0.16	-0.04	0.01	
8-Day	Never	-0.01	0.01	0.09	-0.02	0.002	0.92
	Former	0.005	0.01	0.57	-0.01	0.02	
	Current	-0.02	0.01	0.23	-0.04	0.01	
9-Day	Never	-0.01	0.01	0.05	-0.02	-0.0002	0.79
	Former	0.01	0.01	0.29	-0.01	0.03	
	Current	-0.02	0.01	0.25	-0.04	0.01	
10-Day	Never	-0.01	0.01	0.053	-0.02	-0.0002	0.75
	Former	0.01	0.01	0.20	-0.01	0.03	
	Current	-0.01	0.01	0.32	-0.04	0.01	
11-Day	Never	-0.01	0.01	0.04	-0.03	0.0002	0.74
	Former	0.01	0.01	0.26	-0.01	0.03	
	Current	-0.02	0.01	0.21	-0.05	0.01	
12-Day	Never	-0.01	0.01	0.04	-0.03	-0.001	0.76
	Former	0.01	0.01	0.41	-0.01	0.03	
	Current	-0.02	0.01	0.14	-0.05	0.01	
13-Day	Never	-0.01	0.01	0.03	-0.03	-0.002	0.65
	Former	0.01	0.01	0.42	-0.01	0.03	
	Current	-0.03	0.01	0.06	-0.06	0.001	
2-Week	Never	-0.02	0.01	0.02	-0.03	-0.003	0.61
	Former	0.01	0.01	0.34	-0.01	0.03	
	Current	-0.03	0.01	0.06	-0.06	0.001	
1-Month	Never	-0.03	0.01	0.001	-0.05	-0.01	0.84
	Former	0.01	0.01	0.40	-0.01	0.04	
	Current	-0.02	0.02	0.36	-0.06	0.02	
3-Month	Never	-0.05	0.02	0.002	-0.08	-0.02	0.57
	Former	-0.03	0.02	0.17	-0.06	0.01	
	Current	-0.01	0.04	0.86	-0.08	0.06	
6-Month	Never	-0.05	0.02	0.01	-0.10	-0.01	0.90
	Former	-0.03	0.03	0.29	-0.08	0.02	
	Current	-0.02	0.05	0.66	-0.11	0.07	
1-Year	Never	-0.13	0.04	0.0003	-0.20	-0.06	0.0004
	Former	-0.02	0.04	0.60	-0.11	0.06	
	Current	-0.04	0.07	0.63	-0.18	0.11	
2-Year	Never	-0.16	0.04	<.0001	-0.23	-0.09	0.0002
	Former	-0.05	0.04	0.24	-0.13	0.03	
	Current	-0.09	0.07	0.25	-0.23	0.06	
3-Year	Never	-0.15	0.03	<.0001	-0.22	-0.08	<.0001
	Former	-0.04	0.04	0.38	-0.12	0.05	

Average Variables	Smoking Subgroup	Estimate[a]	Standard Error[a]	P-Value	95% Confidence Limits		P for Interaction
	Current	-0.09	0.07	0.21	-0.22	0.05	

[a] Effect sizes and standard errors are shown per each unit increase in cognitive score.
